# Collective signalling drives rapid jumping between cell states

**DOI:** 10.1242/dev.201946

**Published:** 2023-12-06

**Authors:** Elizabeth R. Westbrook, Tchern Lenn, Jonathan R. Chubb, Vlatka Antolović

**Affiliations:** UCL Laboratory for Molecular Cell Biology and Department of Cell and Developmental Biology, University College London, Gower Street, London, WC1E 6BT, UK

**Keywords:** Cell state transition, Positive feedback, MS2 imaging, Stem cell niche, Oscillation, Transcriptional noise, Optogenetics, Collective behaviour, *In vivo* imaging, Excitable signalling

## Abstract

Development can proceed in ‘fits and starts’, with rapid transitions between cell states involving concerted transcriptome-wide changes in gene expression. However, it is not clear how these transitions are regulated in complex cell populations, in which cells receive multiple inputs. We address this issue using *Dictyostelium* cells undergoing development in their physiological niche. A continuous single cell transcriptomics time series identifies a sharp ‘jump’ in global gene expression marking functionally different cell states. By simultaneously imaging the physiological dynamics of transcription and signalling, we show the jump coincides with the onset of collective oscillations of cAMP. Optogenetic control of cAMP pulses shows that different jump genes respond to distinct dynamic features of signalling. Late jump gene expression changes are almost completely dependent on cAMP, whereas transcript changes at the onset of the jump require additional input. The coupling of collective signalling with gene expression is a potentially powerful strategy to drive robust cell state transitions in heterogeneous signalling environments. Based on the context of the jump, we also conclude that sharp gene expression transitions may not be sufficient for commitment.

## Teaser

**Highlighted Article:** Sharp transitions between successive cell states (‘jumps’) occur during developmental progression. This study shows that jumps can be induced by the emergence of collective signalling in the cell population.

## INTRODUCTION

The changes in gene expression occurring during developmental progression are not constant paced. In diverse developmental contexts, from plants to *Dictyostelium*, to neurons, to adult and embryonic stem cells, developmental progression occurs by rapid and concerted transcriptome-wide switching from one gene expression state to the next (Antolovic et al., 2019; Artegiani et al., 2017; Giri et al., 2022; Jang et al., 2017; Moris et al., 2016; Nelms and Walbot, 2019; Rukhlenko et al., 2022; Saez et al., 2022). These rapid transitions imply a powerful and general mechanism for cells to robustly ‘commit’ to a specific state in the presence of complex tissue signalling, by making cells insensitive to signals promoting alternative states, and by promoting coherence in the establishment of the new state.

Sharp switching between transcriptome states has usually been revealed by single cell transcriptomics methods. Although these approaches allow transcriptomes to be sampled from many cells at a time, and so enable classification of cell states, the measurements require disrupting the cells and their dynamic population structure. Consequently, it is unclear how rapid cell state switching is organised and coordinated in space and time within physiological cell contexts.

Here, we investigate the coordination of rapid cell state transitions using the social amoeba *Dictyostelium.* These cells enter their developmental programme upon exhaustion of their food source. After a few hours of starvation, cells begin signalling to each other using extracellular cAMP, which acts as a chemoattractant and drives the aggregation of the cells into a multicellular mound. Over the next 15-20 h, the mound undergoes a series of morphogenetic transitions, resulting in the generation of the mature final structure – a fruiting body with spores suspended over the substrate by a stalk. In addition to these morphogenetic transitions, the cells change a substantial proportion of their transcriptome as they transition from the feeding state to the final structure. Time series analysis of transcriptomes at the population level reveals, as in other systems, that developmental progression is not constant paced (Parikh et al., 2010; Rosengarten et al., 2015). More recently, single cell transcriptome analysis of the mound stage revealed discrete states during the cell fate bifurcation process, indicating the concerted switching of the transcriptome within single cells (Antolovic et al., 2019).

Gene expression changes are regulated by a variety of signals: the onset of development is regulated by nutritional signalling (Jaiswal and Kimmel, 2019), quorum sensing (Clarke and Gomer, 1995) and cAMP (Cai et al., 2014; Corrigan and Chubb, 2014; Masaki et al., 2013), with other signals operating later during development (Williams, 2006). Despite the involvement of multiple signals during early development, most assays remove this signalling complexity, by plating cells from well-mixed cultures in non-nutrient buffer at uniform density. This removes the natural heterogeneity in developmental time within a *Dictyostelium* colony, and the complex external regulation experienced by each cell is reduced to a time-dependent wait for the onset of cAMP signalling.

To understand cell state switching in a more physiological context, we instead consider the early developmental programme in a mimic of the *Dictyostelium* physiological niche. The cells normally live in the soil, feeding on bacteria, and this is simulated in the lab by plating cells on a lawn of bacteria on an agar plate. As cells clear the bacteria, they create a plaque, in which the starving cells then undergo development. This niche-mimic contains the full asynchronous spectrum of developmental states, and more closely resembles the natural signalling complexity, in which nutrition (bacteria), cAMP and variations in cell density (quorum signalling) co-exist. We contextualise a sharp transition in transcriptome content – the ‘jump’ – which occurs at the transition between the unicellular and multicellular stages of development. The jump emerges as a sharp spatial boundary in the colony as collective cAMP signalling begins. Jump gene expression requires cAMP signalling, however different jump genes respond to cAMP with different dynamic behaviours. Post-jump gene expression is almost completely dependent on cAMP, whereas early jump genes require additional signalling inputs. The jump differentially recruits cells separated by only minutes in developmental time, challenging the standard view of development as a synchronous timer-based process. Based on the context of the jump, we infer that gene expression changes at the jump do not constitute commitment.

## RESULTS

**Fig. 1.**
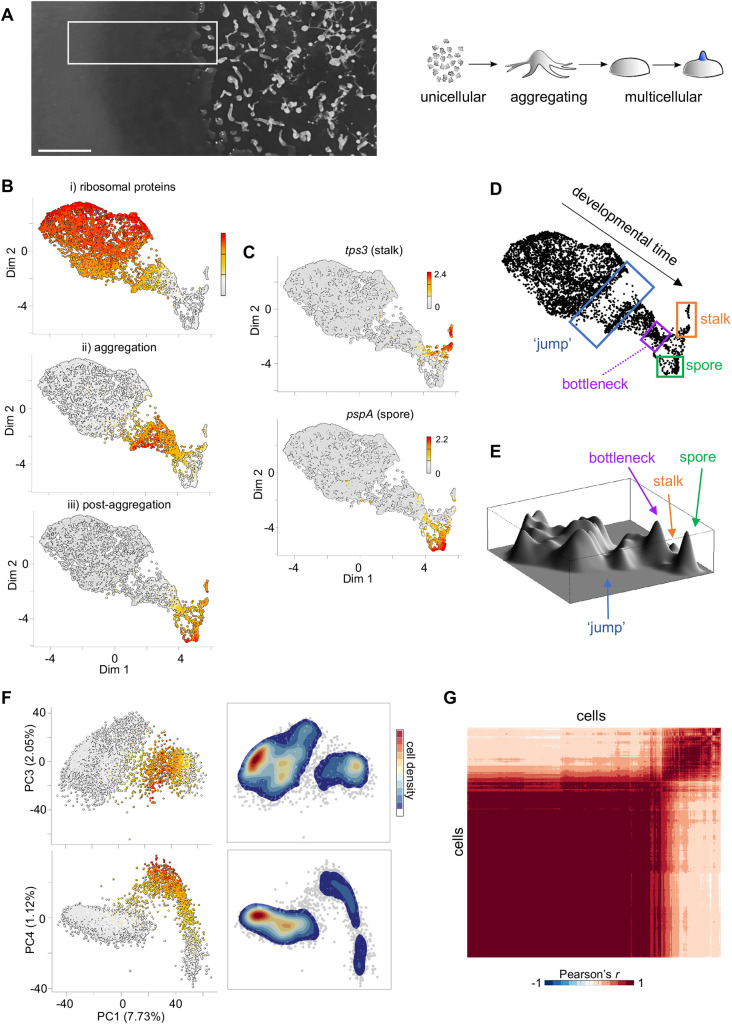
**A jump in developmental progression.** (A) The *Dictyostelium* developmental niche. Left panel: Cells are plated on a bacterial lawn with uncleared bacteria on the left. To the right, bacteria are cleared, cells enter the multicellular state, which goes through morphogenesis to the final fruiting body formation (far right). The white rectangle illustrates the continuous region sampled for transcriptome analysis. Right panel shows a schematic of the sampled life cycle stages. Scale bar: 0.5 cm. (B) 4743 cells positioned in two-dimensional (2D) space, with each cell coloured by the mean expression (mean UMI count) of the following gene sets: (i) ribosomal protein genes (78 genes), (ii) aggregation genes (200) and (iii) genes upregulated in aggregates (215). (C) Expression of stalk (*tps3*) and spore (*pspA*) transcripts in 2D transcriptome space. Scale shows log10 of transcript counts (UMIs). (D) Summary of transcriptome map, showing the jump, bottleneck and cell fate separation. (E) Cell density landscape of D. Landscape ‘height’ represents cell abundance at specific transcriptome states. Few cells are found in the jump region, and cells accumulate in the bottleneck (Fig. S1D). (F) Validation of the jump using PCA. Principal components (PCs) 3 and 4 are plotted against PC1. Each dot is a cell. Colours in left panels are the mean expression level of the aggregation gene set. Colours in the right panels correspond to relative cell density. PC1 approximates developmental progression. Separation of two cell populations (the jump) is clearly visible in both PC1-PC3 and PC1-PC4 space. Aggregation-specific gene expression increases just after the jump (see also Fig. S1E). (G) Two main cell states revealed by a cell–cell correlation matrix, with two distinct clusters visible.

Understanding the regulation of cell state transitions during development requires cell states to be defined in the unperturbed physiological context. To describe the major transitions during developmental progression, we collected a continuous single cell transcriptomics time course of *Dictyostelium* development. To capture development in a continuous manner, we collected cells from colonies of cells feeding on their bacterial food source (Fig. 1A). In this context, cells feed on bacteria, and migrate further into the bacteria to acquire more food. Cells left behind starve, which triggers their developmental programme: single cells aggregate together by chemotaxis towards periodic signalling waves of cAMP, to form mounds. Subsequently, the mound goes through a series of morphogenetic steps, ultimately generating the final structure, with spores suspended above the substrate by a stalk. We collected a continuous streak of cells, from the bacterial zone through to the mounds, then generated single cell transcriptomes for 4743 cells.

To visualise the data, we reduced its dimensionality to two components combining principal component analysis (PCA) and elastic embedding to retain both local and global data structure (Chen et al., 2019). To identify the direction of developmental time within the data, we labelled plots with panels of genes representative of specific stages of development (Fig. 1B): the top plot shows expression of ribosome protein genes, which are strongly expressed during feeding but become repressed during starvation, the middle panel shows expression of aggregation-specific genes and the bottom panel displays expression of genes upregulated after aggregation. Expression of markers of the two principal fates, stalk and spore, occurs in the far right of the plot (Fig. 1C). Overall, these data indicate developmental time proceeds from top left to bottom right along the backbone of the fish-shaped distribution (Fig. 1D). This inferred directionality of developmental time is supported by overlaying expression of an independently generated population transcriptomic dataset (Katoh-Kurasawa et al., 2021) (Fig. S1A,B), and expression of genes with cell cycle control functions, which label clusters in the undifferentiated zone and spore branch, consistent with known cell cycle activity (Muramoto and Chubb, 2008) (Fig. S1C). The distributions of M- and S-phase gene expression are broadly similar, consistent with studies showing *Dictyostelium* lack a G1 phase (Muramoto and Chubb, 2008; Zimmerman and Weijer, 1993).

### Cell state transitions during early development

The distribution of cells in this reduced dimensionality space reveals several key features. As cells differentiate, they encounter a region with few cells – the ‘jump’– indicating a rapid change in the global transcriptome of cells (Fig. 1D). This jump is clearly observed in a 3D density plot of the data (Fig. 1E), where peak heights correspond to cell density in gene expression space. After the jump, cells accumulate at a bottleneck, where their transcriptomes become similar, before undergoing a second rapid transcriptome remodelling, similar to the jump, as they separate into the spore and stalk fates, in agreement with earlier observations (Antolovic et al., 2019). Hierarchical clustering implies cells here proceed from the bottleneck into a mixed transcriptional intermediate state (Fig. S1D, cells marked in purple), in which spore and stalk markers can both be expressed, albeit with little overlap within individual cells, before the complete fate separation occurs.

In this study, we considered the first jump. To test whether this jump is a biological effect or an effect of the non-linear data representation, we also represented the data using linear dimensionality reduction: PCA. In PCA, the PC1 axis reflects developmental time (Fig. S1E), with the jump clearly apparent in several higher order principal components (Fig. 1F, Fig. S1E), indicating it is not an artefact of the elastic embedding procedure. The jump is also clear in clustering of cell–cell Pearson correlations (Fig. 1G), which reveals two major clusters, corresponding to the cells before and after the jump.

To gain insight into the gene expression changes occurring during the jump, we carried out unbiased hierarchical clustering on the whole dataset. The clustering revealed the sharp changes in global expression profiles occurring during the jump and identified four major clusters (Fig. S1F, top panel), which are highlighted on the 2D embedding plot (Fig. S1F, bottom panel): two clusters of cells before the jump and two after the jump. Based on gene expression signatures, these clusters represent cells that are feeding (red), starving (green), aggregating (blue) and mound stage (purple). Our inference here, also apparent in Fig. S1A, is that the jump occurs at the onset of aggregation. This is consistent with population transcriptomic data based on morphologically staged time series that show substantial transcriptome changes between single cell and multicellular stages (Katoh-Kurasawa et al., 2021; Parikh et al., 2010). However, the implication from our continuously sampled data showing few cells caught within the jump is that the transition is concerted within individual cells, with two clearly demarcated attractor states (Fig. 1D-F), features not resolvable using population average data. The majority of changes before the jump are repressive, with 66% of transcripts downregulated in two waves before the jump (Fig. S1F). Transcript clearance might result from transcriptional repression followed by constitutive RNA turnover, or by induced RNA decay. Concerted transcriptome shifts within a cell, based on transcriptional repression, would require the half-lives of repressed transcripts to be matched, to enable synchrony. This is not consistent with data showing a broad heterogeneity in turnover times for different mRNAs during starvation (Muramoto et al., 2012), implying the jump requires an induced RNA turnover mechanism.

**Fig. 2.**
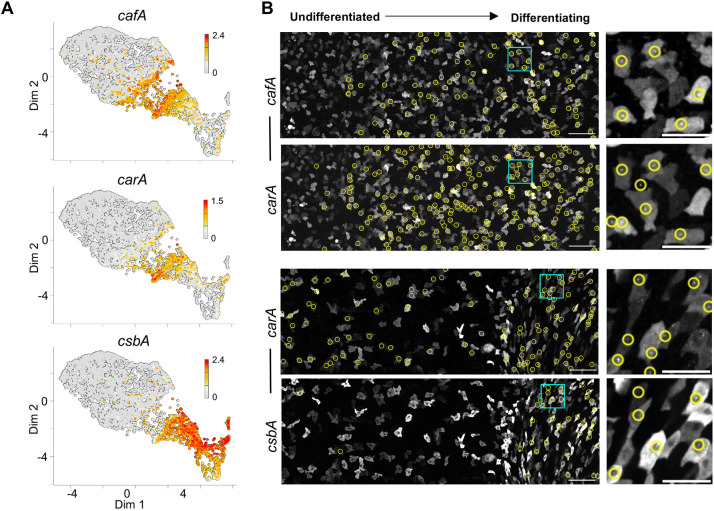
**Matching jump transcripts to niche context.** (A) Expression of specific genes around the jump. 2D transcriptome maps are coloured by the expression level of the indicated genes. Scale shows log10 of transcript counts (UMIs). (B) Imaging nascent transcription in the developmental niche of genes that change expression during the jump. On the left are the undifferentiated cells, on the right are cells beginning to show collective chemotaxis. Top two panels show transcription of *cafA*-PP7 and *carA*-MS2 in the same cells. Yellow rings highlight cells with spots corresponding to nascent transcription. Bottom two panels show transcription of *carA*-MS2 and *csbA*-PP7, in the same cells. Boxed areas are enlarged on the right. Scale bars: 50 µm (left); 20 µm (right).

To contextualise the jump with developmental progression more precisely, we used live imaging of transcription of jump marker genes, using transcriptional reporters inserted into endogenous gene loci. We identified jump markers in the transcriptome data that are representative of cells at different stages of the jump (Fig. 2A). The *cafA* gene, which encodes a calcium-binding protein, is induced prior to the jump. *carA*, the cAMP receptor gene, is expressed slightly later, with detectable induction before the jump. The *csbA* gene, which encodes a cell adhesion protein, is expressed post-jump. To directly visualise transcription of these markers during development, we inserted MS2 (Bertrand et al., 1998) and PP7 (Larson et al., 2011) stem loops into the endogenous gene loci, then used the cognate fluorescent MCP and PCP stem loop binding proteins to visualise nascent transcripts as spots at the site of transcription (Fig. 2B) (Tunnacliffe and Chubb, 2020). Genes were imaged in pairs, with simultaneous imaging of both MS2- and PP7-tagged genes, with *carA*-MS2 imaged alongside both *cafA-*PP7 and *csbA-*PP7 to benchmark the spatial context of expression for each gene. To ensure physiological regulation, and to contextualise transcription with normal developmental progression, cells were directly imaged in the developmental colony. Both *cafA* and *carA* were strongly induced in cells before the onset of cell aggregation*.* In contrast, *csbA* only showed abundant transcriptional events in the zone of the colony undergoing aggregation.

### Regulation of jump gene expression by cAMP

**Fig. 3.**
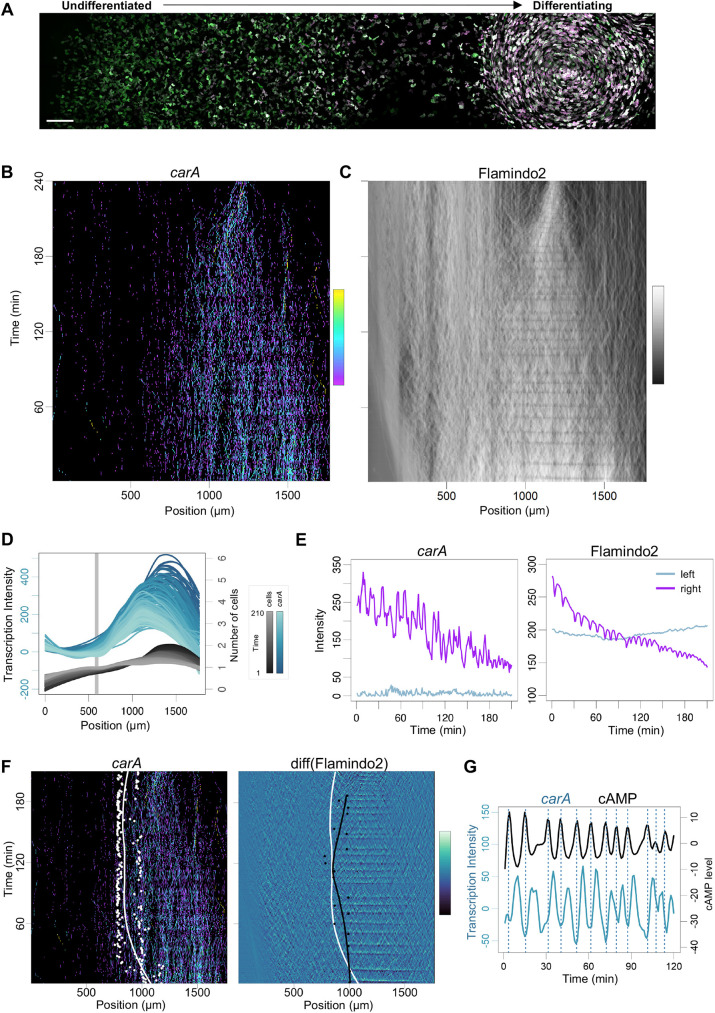
**Coupling between jump transcription and signalling dynamics.** (A) Overview image of the *Dictyostelium* early developmental niche. Undifferentiated, feeding cells are on the left, becoming progressively more differentiated to the right and entering a multicellular aggregate on the far right. Scale bar: 100 µm. (B) Imaging transcription dynamics of the jump gene *carA* in the niche. Horizontal axis reflects the axis of differentiation in A. The vertical axis is imaging time. Transcription spot intensity over time is shown, with activity level related by the colour scale bar [yellow (high) to purple (low)]. Transcription is sporadic in the less differentiated cells, becoming frequent and oscillatory as differentiation proceeds. Transcription spot intensities were averaged into 10 pixel bins (10×0.35 µm). (C) Same data as in B, showing cAMP signalling using the Flamindo2 biosensor, which dims in fluorescence upon binding cAMP. Data show oscillations in differentiating cells. Cells merge into an aggregate towards the end of the time series. (D) Increased transcription activity during differentiation. Plots summarise the data in B, and also show the distribution of cells in the population. Changing transcription and cell distributions over time are shown as different colour shades (see colour scale). The grey line corresponds to the minimum in cell density, where the population splits during the transition to multicellularity. (E) Transitions in transcription and signalling dynamics across the niche. Left panel shows distinct *carA* transcription dynamics comparing zones left and right of the grey line in D. Right panel shows the distinction between oscillatory and non-oscillatory cAMP dynamics either side of the grey line. (F) Positional coupling between transcription and signalling dynamics. Left: White spots are inflections of the curves of transcriptional intensity values at each imaging time point. The white line is a regression line summarising the distribution of points. Right: Black dots show inflection points for cAMP signalling, with the black line the regression line and white line the same as in the left panel. Inflection values were calculated at time points of cAMP wave maxima. Diff(Flamindo2) represents the difference in intensity between one time point and the subsequent one. (G) Temporal coupling between transcription and signalling oscillations. Peaks in cAMP signalling (vertical lines) occur 4-5 min prior to peaks in *carA* transcription.

Developmental gene expression can be influenced by multiple signals, notably starvation time (Jaiswal and Kimmel, 2019) and extracellular cAMP (Cai et al., 2014; Corrigan and Chubb, 2014). To what extent are these signals, which are spatially heterogeneous in the niche, driving the gene expression changes at the jump? As both transcriptomics and the imaging imply jump genes such as *cafA* and *carA* are induced just prior or at the onset of aggregation, this suggested cAMP signalling may be responsible for the jump. To test this, we imaged transcription of jump genes in the colony (Movie 1), with parallel tracking of cAMP signalling, by mixing the transcriptional reporter cells with cells expressing the cAMP reporter Flamindo2. Flamindo2 is an intensiometric cAMP reporter that dims in fluorescence when it binds to cAMP (Ford et al., 2023; Hashimura et al., 2019; Kundert et al., 2020) (Movie 2). We obtained time series data simultaneously recording the physiological dynamics of both transcription and signalling, in the unperturbed colony, from the undifferentiated cells through to the cells at the aggregation stage of differentiation, over millimetre length scales (Fig. 3A). Data are represented with the horizontal axis representing the position of each cell in the colony (Fig. 3B). Undifferentiated cells are on the left, with the differentiating cells on the right. The vertical axis represents imaging time. For *carA*, the undifferentiated cells only showed sparse and sporadic transcription (Fig. 3B, Fig. S2A), with transcription becoming strong and oscillatory in the more differentiated cells. The region of strong transcription coincided with the domain of cAMP fluctuations, which showed oscillatory behaviour (Fig. 3C-E, Fig. S2B-D), and continued as the signalling cells merged into an aggregate towards the end of the time series (observed as the constriction of fluorescence at the top right of Fig. 3C). The *carA-*expressing cells mark the zone in the colony where aggregating cells peel away from the rest of the population: the population that was spatially continuous at the onset of imaging separated gradually as the aggregate formed.

To what extent are the oscillations temporally and spatially coupled? A quantitative analysis of *carA* transcription indicated *carA* induction occurs at the same region at which cAMP relay is occurring. This was revealed by substantial overlap in the inflections of the curves summarising transcription and signalling activity (Fig. 3F, Fig. S2E). The period of the cAMP and *carA* transcription oscillations was similar, however the phases of transcription and signalling waves were offset (Fig. 3G, Fig. S2F). A cross-correlation analysis revealed a lag of 4-5 min between the peak of the cAMP wave and the peak of transcription, possibly reflecting signalling lags from receptor to gene, such as transcription factor shuttling times (Cai et al., 2014), in addition to the time for transcripts to build up at the locus. Overall, these data imply induction of *carA* by collective cAMP signalling.

**Fig. 4.**
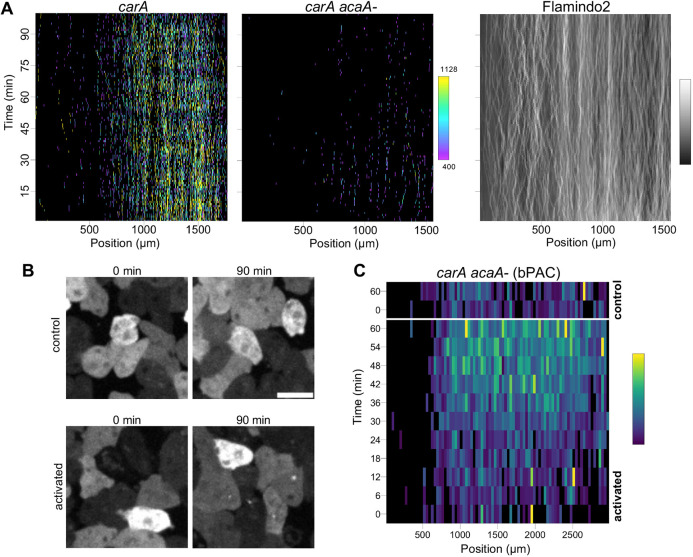
**Functional coupling of cAMP signalling and jump gene expression.** (A) Loss of *carA* transcription in cells lacking a functional adenylyl cyclase A (ACA) gene. Left panel shows the rapid increase in *carA* transcription in wild-type cells (expanded view of Fig. 3B). Central panel shows loss of *carA* induction in *acaA-* cells. Right panel shows absence of cAMP oscillations in *acaA−* cells. Typical experiments shown. Three wild-type and three *acaA−* biological repeats carried out. (B) Optogenetic rescue of jump gene expression: *acaA− carA-*PP7 cells mixed with *acaA−* cells expressing optogenetic adenylyl cyclase, bPAC. Cells were pulsed with blue light at 6 min intervals to mimic normal cAMP signalling. Strong induction of transcription was observed in pulsed cells (bottom) compared to non-pulsed cells (top). Scale bar: 10 µm. (C) *carA* shows context- and time-dependent responses to exogenous induction of cAMP using bPAC. Heatmap shows *carA* induction in the cell population after 30 min of pulsing, but not close to the undifferentiated zone. Transcription spot intensities were averaged into 100 pixel bins (100×0.35 µm). Typical experiment is shown from three repeats (two biological).

To directly test the role of cAMP in inducing jump transcription, we imaged *carA* transcription together with signalling in *acaA−* mutants (Fig. 4A), which lack the adenylyl cyclase that synthesises cAMP during early development. These mutants showed a loss of cAMP signalling using the Flamindo2 reporter (Fig. 4A, right panel). The rare sporadic *carA* transcriptional events were still observed, with slightly enhanced activity further from the bacterial zone, however the gene did not show the strong induction of transcription observed in the wild-type developmental collective.

To test to what extent cAMP signalling is sufficient to induce jump gene expression, we exposed cells across the colony to periodic pulses of cAMP using the optogenetic adenylyl cyclase bPAC from the soil bacterium *Beggiatoa* (Stierl et al., 2011). To effectively control the experiment in the absence of exogenous cAMP signalling, we used *acaA−* cells, to prevent cAMP signals propagating across the colony, and to allow test (activated) and control (not-activated) cells to be compared in the same conditions. We activated bPAC at 6 min intervals with blue light pulses along the entire starvation axis of the developmental collective. This pulse frequency was used to mimic the normal excitable cAMP signalling pulses occurring around the onset of cell aggregation. This regime of pulsing caused the induction of bright *carA* transcription spots in activated cells, but not in the ‘no light’ controls (Fig. 4B). When examined over the whole collective, the induction process revealed other features of the niche that influence cell responsiveness (Fig. 4C). Firstly, the induction was not immediate – the cells required around 30 min of pulsing before showing strong induction, indicating some requirement for priming. Secondly, the induction was spatially restricted, with the less-differentiated cells at the left of the colony not showing *carA* induction, implying repression by some feature of cell context in this zone. So, although these data indicate oscillatory cAMP signalling drives the jump, the responsiveness of cells depends on their context in the niche.

### Jump genes have different regulatory inputs

**Fig. 5.**
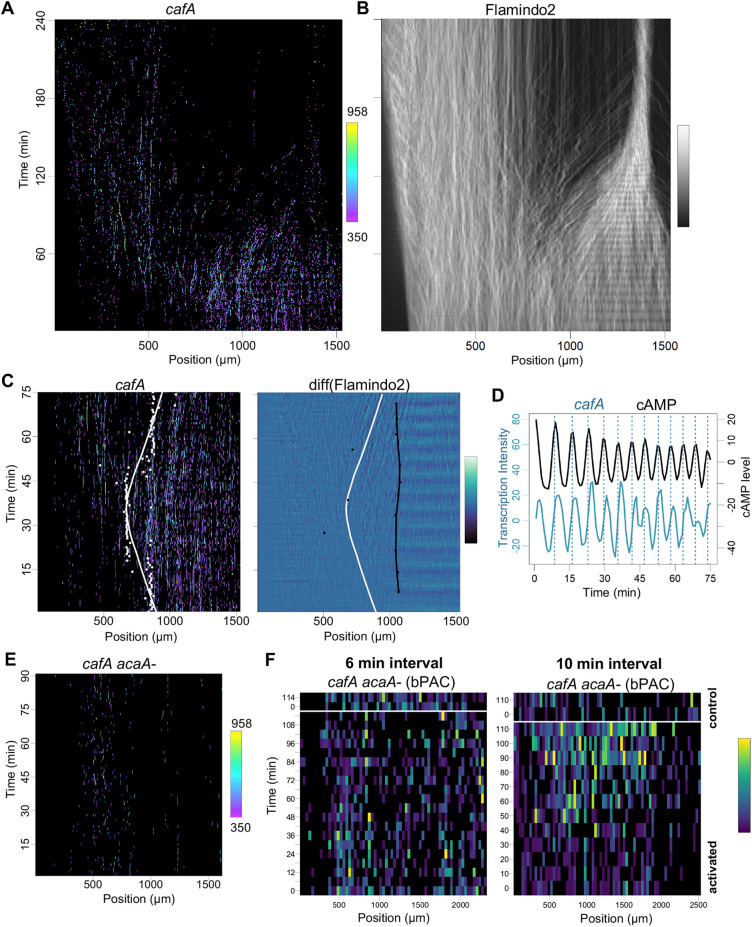
**Alternative coupling strategies between jump signalling and transcription.** (A) Imaging transcription dynamics of the jump gene *cafA*. Horizontal axis reflects the axis of differentiation. Vertical axis shows imaging time. Transcription is sporadic in the less differentiated cells, becoming frequent and oscillatory during differentiation. (B) Same cell field as A, showing cAMP signalling dynamics. Cells merge into an aggregate during the movie. (C) Non-overlapping boundaries of transcription and signalling. Left: White spots represent inflections of the curves of transcriptional intensity values at each imaging time point. The white line is a regression line summarising the distribution of points. Right: Black dots show inflection points for cAMP, black line is the regression for these points. White line the same as in left panel. (D) Temporal coupling between transcription and signalling. Peaks in cAMP signalling slightly precede peaks in *cafA* transcription. (E) Loss of *cafA* transcription in cells lacking a functional adenylyl cyclase A (ACA) gene. Typical experiments are shown in A and E from seven wild-type and four *acaA−* (biological repeats). (F) Left: Optogenetic activation of cAMP with a 6 min pulse interval does not rescue *cafA* gene expression. *acaA− cafA-*PP7 cells mixed with *acaA−* cells expressing optogenetic adenylyl cyclase, bPAC. Cells were pulsed with blue light at 6 min intervals to mimic normal cAMP signalling around aggregation onset. Unlike for *carA*, induction of transcription was not observed in pulsed cells. Typical experiment is shown from nine repeats (five biological). Right: Induction of *cafA* transcription by optogenetic cAMP pulses with a 10 min interval. Strong induction was observed in 3/8 replicates. Transcription spot intensities were averaged into 100 pixel bins (100×0.35 µm).

Temporal coupling was also observed between cAMP oscillations and the *cafA* gene (Fig. 5, Fig. S3), however *cafA* showed different behaviour compared to *carA*. Transcription of *cafA* was observed in areas of the cell population without oscillatory cAMP signalling, although stronger transcription was observed in the zone where cAMP oscillations were detected (Fig. 5A-C). The transcription was also oscillatory, however, unlike *carA*, the gene was repressed at the higher cAMP oscillation frequencies occurring later in the time series (Fig. 5A, Fig. S3A). A further difference between *carA* and *cafA* was apparent in the offset between transcription and signalling, with *cafA* transcription maxima delayed from cAMP maxima by 90 s or less (Fig. 5D, Fig. S3F).

To directly test the role of cAMP in inducing *cafA* transcription, we imaged *cafA* transcription and cAMP signalling in *acaA−* mutants (Fig. 5E). The rare sporadic *cafA* transcriptional events were still observed. However, the gene failed to show the strong induction of transcription normally observed in wild-type cells. Unlike *carA*, the *cafA* gene was not induced by optogenetic pulses of cAMP synthesis with a 6 min periodicity (Fig. 5F, left panel). Therefore, although strong induction of both genes requires cAMP, *carA* and *cafA* show distinct kinetics of coupling to cAMP signalling. Our observations that *cafA* transcription is repressed at high cAMP frequencies (Fig. 5A), together with the observed induction of transcription in cells not undergoing robust cAMP oscillations (Fig. 5A,C) suggest *cafA* may respond to lower levels of cAMP and/or lower frequency pulses, with repression of the gene at high signal amplitudes/frequencies. To explore this further, we repeated the optogenetic activation experiments for *cafA* transcription, this time using a 10 min pulse interval (Fig. 5F, right panel). With this reduced cAMP pulse frequency, we observed induction of *cafA* transcription in the niche. The induction was not fully penetrant, with strong transcription induced in three out of eight experimental replicates, however the results are suggestive that the *cafA* gene is responsive to cAMP, but at lower amplitudes or frequencies of stimulation. This would be consistent with its expression earlier in development than *carA*, when cAMP signalling is more unstructured and infrequent (Ford et al., 2023).

The coupling of *cafA* transcription to cAMP may follow the rules inferred for the transcriptional oscillations of the *csaA* gene (Cai et al., 2014; Corrigan and Chubb, 2014). With the caveat that *csaA* oscillations were observed with cells differentiating in buffer, rather than in the niche, the gene was proposed to show two-step regulation, with activation and repression at different stages of the cAMP oscillation cycle. The effect of this scenario is that the gene is switched off at high cAMP wave frequencies, as the repression occurs before the activated state has sufficient time to be productive. Transcription of *cafA* is repressed at high cAMP frequencies (Fig. 5), in addition to showing activation independent of cAMP oscillations, much like *csaA*. In contrast, the *carA* gene was not inactivated at high signal frequencies (Fig. 3), suggesting a more simple one-step model, in which the gene activation mirrors the level of cAMP (with a lag) but no explicit repressive input.

We then further tested the requirement for cAMP signalling for a broad set of genes changing their expression at the jump. To define this set of genes, we intersected high temporal resolution population transcriptomic datasets from synchronous developmental protocols (Katoh-Kurasawa et al., 2021) with our own continuous single cell transcriptome data from the physiological colony (Fig. S4). We categorised genes into three profiles (Table S1): repressed at the jump (pre-jump genes), induced spanning the jump (jump genes such as *carA* and *cafA*) and induced after the jump (post-jump). Comparing the population transcriptomic data for wild-type and *acaA−* cells revealed effects of cAMP removal on all three categories. For pre-jump genes, 85% failed to be repressed without cAMP signalling (Fig. S4B; 46/54 genes). For jump genes, 16/22 showed partially reduced expression without cAMP, with the remainder losing induction completely (Fig. S4C). Post-jump genes almost entirely showed complete lack of normal developmental expression without cAMP, with only 1/82 genes (*csbC*) retaining detectable induction (Fig. S4D). Overall, these data indicate that erasure of the undifferentiated state requires cAMP and the post-jump state is effectively absent without cAMP. In contrast, as also implied by the sporadic transcription observed in our niche imaging, and the resistance to optogenetic cAMP stimulation we observed for recently starved cells, induction of the transcripts spanning the jump state requires a mixture of cAMP signalling and other inputs.

### Collective signalling separates cells of a similar developmental age

**Fig. 6.**
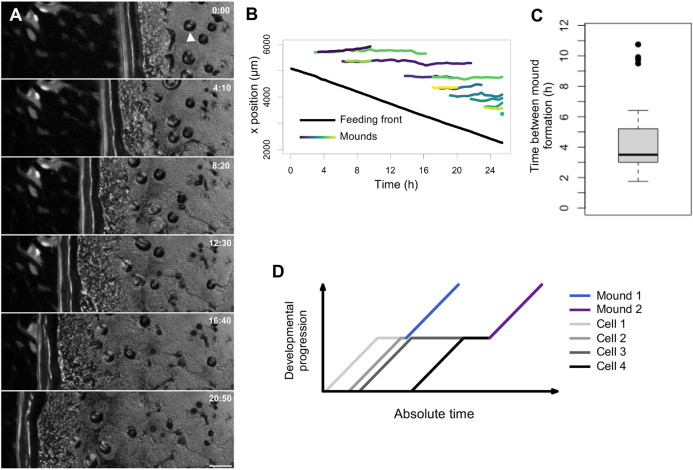
**Aggregation combines cells with widely different developmental times.** (A) Cell deposition by the advancing feeding front. Stills from a time-lapse movie show cells clear the bacterial lawn at a constant rate from right to left. Behind the cleared lawn cells are left behind, which aggregate into mounds at discrete steps. Scale bar: 0.5 mm. Time shown in h:min. Arrowhead indicates an example mound. (B) Quantification of the movie in A showing feeding front progression (black line) and individual mound formation events in the recently starved zone. For clarity, the data are extracted from the bottom half of each panel in the movie. Different mounds shown as different colours. (C) Time between mound formation from 150 pixel sections (0.63 mm) of the feeding front. Data shown as a box plot, showing the median (line), interquartile range (IQR; box), minimum and maximum values excluding outliers (whiskers). Outliers are defined as 1.5× the IQR above or below the box. (D) Schematic showing scenarios caused by discrete budding events. Cells are continuously shed from the feeding front. Each line represents an example cell that leaves the feeding front from this continuously shed population. Cell 1 leaves the front and forms a mound with Cell 2. Cell 3 buds shortly after Cell 2 but waits a long period to enter a mound with Cell 4.

In the developmental niche, cells that peel off to join streams of aggregating cells are initially spatially directly adjacent to cells of a similar developmental time (e.g. Fig. 3B). To quantify this, we captured low-magnification time series of the developmental colony (Fig. 6A). The cells advanced into the bacterial zone at a constant rate of around 1.9 µm min^−1^, which is slightly slower than they migrate *in vitro* in buffer (Chubb et al., 2002). The events in which cells peeled off to form streams and then mounds occurred around once every 4 h (Fig. 6B,C) although this could be as much as 10 h. This may be an underestimate of the variability, with rare mounds forming well behind the normal band of mound formation, in the zone containing fruiting bodies. Overall, this variation implies the absolute time of starvation, which reflects the continuous clearance of the bacteria away from the starving cells, is not a precise predictor of the time at which the cell enters multicellular development, which is a discrete event. As a consequence, cells entering mounds will vary in developmental time by the size of the interval between peel-off events. To contextualise this variation in timing, the normal starvation time before aggregation onset in synchronous developmental protocols is 4-6 h, depending on the strain used, and standard experimental variation. As a result, cells entering late into a mound will have experienced around two-fold (or sometimes considerably more) extra nutrient deprivation than cells early into a mound (schematic in Fig. 6D). This represents a substantial spontaneous heterogeneity in cell signalling history, which may underlie the observed sub-clustering of cell gene expression states just before the jump (Fig. 1E). This heterogeneity may have functional consequences: nutrient-deprived cells tend to adopt the stalk rather than spore fate (Thompson and Kay, 2000). The spontaneous formation of mixed-age mounds by the jump would therefore provide a straightforward source of nutritional heterogeneity to facilitate robust cell type patterning.

## DISCUSSION

There are key features of cAMP signalling that well suit its ability to drive a sharp change in cell state. As with many tissue signalling processes in more complex systems (Deneke and Di Talia, 2018; Dieterich et al., 2006; Ender et al., 2022; Liu et al., 2022; Pond et al., 2022), signalling by cAMP is excitable: as one cell is activated, it releases more signal to its neighbours, which then further spread the signal (Ford et al., 2023; Gregor et al., 2010; Tomchik and Devreotes, 1981). This signal relay will enable coordinated switching of a cell population into the new state, necessary for an organised response. In addition, the genes induced at the jump, as exemplified by *carA* (which encodes the cAMP receptor) provide the potential for positive feedback. The ability of a signal to induce its own receptor, in addition to the induction at the jump of other genes required for cell aggregation, will further strengthen cAMP signalling between cells. This mutual interaction allows an amplification ideally suited to rapidly lifting a cell out of one state and into the next.

One consideration is that cells would need to be able to perceive cAMP to get the amplification process started, which will require a cAMP receptor. Consistent with this requirement, feeding cells can show basal levels of expression from the *carA* locus (Muramoto et al., 2012), so there will be the potential to detect early arriving cAMP. A further issue is that although induction of transcript clearance and post-jump transcription appear dominated by cAMP regulation, the induction of most genes spanning the jump, notably *cafA*, is also modulated by other inputs. This makes regulatory sense – for a cell to embark on a sharp state transition, multiple inputs would provide more robustness to this decision. Overlying a collective signal over a timing mechanism (starvation) means the cell will only jump when there is a sufficient quorum to make the transition to multicellularity worthwhile, whilst allowing sufficient time to not miss out on another opportunity to feed.

Sharp state transitions or jumps have been implicated as ‘commitment’ points (Mulas et al., 2021). Definitions of commitment vary, but a standard usage implies some resistance against cells reverting to their former state. This usage may not apply to the jump we are considering here. Differentiating *Dictyostelium* cells can de-differentiate rapidly in response to the reapplication of their nutrition source (Finney et al., 1987; Nichols et al., 2020). De-differentiation of most cells in the population is complete within no more than a day and cells retain the ability to de-differentiate until they terminally differentiate, many hours after the jump. This indicates the jump itself presents no absolute barrier to cell state reversion. However, de-differentiation is usually induced by experimental disaggregation of developing structures. If nutrition is applied to intact structures, or cells around the onset of multicellularity, they de-differentiate poorly, if at all (Katoh et al., 2007). This resistance to de-differentiation can be considered commitment but is likely to result from the stability of the signalling across networks of cells, rather than any stable cell-autonomous state resulting from the jump. Indeed, mutant cells which generate unstable mounds show signatures of de-differentiation (Katoh-Kurasawa et al., 2021), suggesting the differentiating state is stabilised by cell interactions, not directly by gene expression state. Does this relate to cell state transitions in general? To an extent, perhaps. Ground state mouse embryonic stem cells can populate preimplantation blastocysts with high efficiency, yet slightly more differentiated cells can reset with a low frequency to contribute to chimeras, although most are lost by cell competition (Alexandrova et al., 2016). Another consideration is that mammalian development requires much more time, and perhaps more cell state transitions than *Dictyostelium* development, so developmentally advanced cells may no longer have the machinery to interpret the signals promoting an earlier state, meaning full de-differentiation can only be enabled by more aggressive approaches, such as forced transcription factor expression.

The single cell gene expression data reported here reveal unexpected sources of cellular heterogeneity. Although the effects of dimensionality reduction also need to be considered, the cells appear heterogeneous in the feeding state before becoming more heterogeneous prior to the jump. This increase in heterogeneity was previously observed in cells differentiating in buffer, which was suggested by modelling to result from the effect of transcription repression on transcriptional noise (Antolovic et al., 2017). Starving cells reduce their overall transcriptional output (Mangiarotti et al., 1981), as might be expected in a context opposed to extensive biosynthesis, which may provide the driver for the increased noise. We show here that there is another potential layer of heterogeneity arising from differences in starvation time of cells undergoing the jump. This heterogeneity could conceivably contribute to variable responses of cells to signals later in development. Indeed, based on the effects of experimental nutrition deprivation on perturbing cell fate allocation (Thompson and Kay, 2000), this spontaneous heterogeneity in nutritional history for cells entering the multicellular stage may contribute to the overall fate diversity between cells in the final developed structure. Input to fate choice will also likely include differences in cell cycle position, which can be a functional source of heterogeneity for cell type allocation in *Dictyostelium* (Gomer and Firtel, 1987; Thompson and Kay, 2000) and other differentiation systems (Pauklin and Vallier, 2013).

Although the pre-jump heterogeneity is largely consistent with the long-held notion that fate choice during development requires differences between cells in feeding and starvation, it is not clear why this heterogeneity should then become reduced before the onset of fate marker expression – the bottleneck. This constriction of cell variability resembles previous single cell transcriptome measurements in the mound (where cell fate bifurcation first becomes detectable), which identified a compact population before the branching into spore and stalk trajectories (Antolovic et al., 2019). The most likely explanation is that the cells at this stage are aggregating or recently aggregated and, regardless of their final fate, will be challenged with expressing the components required for enacting the single cell to multicellular transition. These transcripts dominate the measured transcriptome and are shared by all cells (Antolovic et al., 2019). Based on the sensitivity of single cell transcriptomics, these might be expected to obscure the more variable transcripts conveying information to cells for fate allocation. Alternatively, the cell–cell differences required to inform fate may be better represented in the proteome.

## MATERIALS AND METHODS

### Cell handling

Cells were cultured on lawns of *Klebsiella pneumoniae* on plates of SM agar (Urushihara, 2006)*.* For transcriptomics, we used the wild isolate strain NC4 (from Pauline Schaap, University of Dundee, UK). For genetic modifications, we used the Ax3 strain, and an Ax3 derivative expressing the nuclear marker, H2Bv3-mCherry under the control of the endogenous *rps30* promoter (Corrigan and Chubb, 2014). All cells were used from a master stock and cultured for no more than 10 days. For DNA transformations, we used an electroporation protocol based on H50 buffer (Paschke et al., 2018), with selections in standard HL5 axenic growth medium at 22°C, in tissue culture dishes. Selection used 20 µg/ml G418 for extrachromosomal expression vectors and either 10 µg/ml blasticidin S or 35 µg/ml hygromycin for gene-targeting vectors.

### Molecular biology

To image transcription, PP7 repeats were inserted into endogenous *cafA*, *carA* and *csbA* genes. For PP7 targeting with PP7 cassettes, fragments containing 24 PP7 repeats (Larson et al., 2011) and a blasticidin S resistance (*bsr*) gene (Faix et al., 2004) were inserted at the junction of the promoter and coding sequences of the genes, slightly downstream of the translation start codon. For *carA-*PP7, we used the *carA-*MS2 targeting vector described previously (Muramoto et al., 2012), and replaced the BamHI fragment containing MS2-*bsr* with a BamHI fragment containing PP7-*bsr*. For *cafA-*PP7, we generated a targeting vector with targeting arms cloned as follows: −297 to +281 (promoter, with +1 marking the ATG), +284 to +1310 (coding sequence); for *csbA*: −373 to +274 and +288 to +1148, with HindIII and BsrGI used for cloning promoters, and SpeI and NotI for coding sequences, with PP7-*bsr* inserted using BsrGI and SpeI. Dual transcriptional reporter cell lines with *carA*-MS2 and either *cafA-*PP7 or *csbA-*PP7 were generated in Ax3 *carA*-MS2 knock-in cells (Muramoto et al., 2012) pre-modified by Cre recombinase expression to remove the *bsr*. Single reporter lines for *carA-*PP7 and *cafA-*PP7 were generated in H2Bv3-mCherry-labelled cells. Labelling of the MS2 and PP7 repeats was enabled by expression of extrachromosomal plasmids expressing GFP- or TdTomato-tagged MCP and PCP stem loop binding proteins (Antolovic et al., 2019). For stable uniform Flamindo2 expression, we targeted a codon-optimised Flamindo2 gene into the *act5* gene of Ax3 cells as previously described (Ford et al., 2023). To disrupt the *acaA* gene, we used hygromycin-selectable *acaA* targeting vector (Tweedy et al., 2020). For bPAC, a codon-optimised bPAC gene (Ford et al., 2023) was expressed from the extrachromosomal vector pDM1203 (Paschke et al., 2018), in Flamindo2-expressing *acaA−* cells. All plasmids and cell lines will be deposited at dictyBase.

### Single cell transcriptomics

For a continuous scRNAseq time course, we took a scrape of feeding fronts of NC4 cells, from inside the bacterial zone through to the mound stage of development. Cells were inoculated into ice-cold KK2 buffer (20 mM KPO_4_, pH 6.0), and disaggregated by gentle pipetting. To remove bacteria, cells were centrifuged at 720 ***g*** for 2 min, then resuspended in ice-cold KK2. Single cell transcriptomes were derived using the Chromium Single Cell A Chip platform (PN-1000009) based on a previously published protocol (Nichols et al., 2020). Detailed information on sequencing, downstream processing and data analysis is in the Supplementary Materials and Methods. Transcriptomes from 2671 and 2072 cells, from two replicates, were used for further analysis. Sequencing data are available in the Gene Expression Omnibus database under accession number GSE220242. Code for scRNAseq data analysis is available at https://github.com/Vlatka22/scRNAseq_Pipeline.

### Imaging and image analysis

For imaging gene activity with signalling, transcriptional and signalling reporter cells were mixed at a 1:2 ratio, and spotted onto lawns of *Klebsiella* on diluted SM agar plates (1 SM:19 H_2_O). After 3 days for colonies to form, agar pads were excised and inverted onto imaging dishes (µ-Dish, Ibidi, 81156). Imaging used an inverted spinning disc confocal microscope (3i) using a 63× oil lens, with a Prime 95B CMOS camera (Photometrics). We captured 14-16 *z*-slices, with a 0.4 µm step size and 2×2 binning. 3D stacks were captured every 45 or 60 s at multiple *xy* positions across the cell population, with fields of view stitched to generate a complete view of the early developmental niche. GFP and mCherry/TdTomato were excited with 488 nm and 561 nm lasers, respectively, with laser powers optimised for best resolution alongside maintained cell health. For bPAC activation, transcriptional reporter cells were mixed 1:2 with bPAC-expressing cells. Activating bPAC used a 3D stack with a 488 nm laser every 6 or 10 min. This illumination had the dual function of activating bPAC and collecting transcription spot data.

For low-magnification imaging of feeding front dynamics and mound formation, we spotted Ax3 cells on bacterial lawns on 1:5 diluted SM agar plates, allowed 3 days for colonies to form then captured images every 5 min for up to 25 h. Images were captured using a Dino-Lite digital microscope version 2.0 in a humidified chamber. We tracked the *x* position of the feeding front and mound position every five frames.

Spot detection was based on the approach of Corrigan et al. (2016). To identify cAMP waves, we masked signal from the transcriptional reporter cells, which are more variable in their background intensity than the Flamindo2 cells. The intensity of the remaining cell-containing pixels (representing primarily the Flamindo2 signal) was averaged at each time point. Detailed analysis protocols and methods to compare signalling and transcription distributions are described in the Supplementary Materials and Methods, including minimal modifications to the code from Corrigan et al. (2016). Image analysis code for processing steps downstream of spot detection (with links to spot and signal intensity data) is accessible at https://github.com/Vlatka22/ImageData_Analysis.

## Supplementary Material

10.1242/develop.201946_sup1 Supplementary information

Table S1. The table lists three classes of genes which change their expression around the jump: repressed at the jump (pre-jump genes), induced spanning the jump (“jump” genes) and induced after the jump (post-jump). These gene sets form the basis of the analysis in Supplementary Figure 4.
