# Supplementary material for: Collective signalling drives rapid jumping between cell states
Source: Development. 2023 Dec 6;150(23):dev201946. doi: 10.1242/dev.201946 (PMC10730084; doi:10.1242/dev.201946)
Supplement: Supplementary information [file develop-150-201946-s1.pdf]

Supplementary Materials and Methods

Single cell transcriptomics

For single cell RNAseq of a continuous developmental transition, we took a scrape of a feeding front of NC4 cells, from inside the bacterial zone through to the mound stage of development. Cells were immediately inoculated into ice-cold KK2 buffer (20mM KPO_4, pH 6.0), and disaggregated by gentle pipetting. To remove the bacteria, cells were centrifuged at 720g for 2 minutes, then resuspended in ice-cold KK2. Single cell transcriptomes were derived using a Chromium Single Cell A Chip platform (PN-1000009), with sequencing carried out using a NextSeq500 Mid-output 150-cycle kit. The protocols were as described previously (Nichols et al., 2020), with one modification, 12 and 13 cycles were used for sample index PCR of the replicates, rather than 11 and 12.

Alignment, barcode counting, UMI counting, and filtering was performed by Cell Ranger v3.1.0 using default parameters. For the two biological replicates, totals of 2925 and 2404 single cell libraries passed the filter, with a median of around 15000 and 13000 transcripts per cell (unique molecular counts: UMIs), and 2200 and 2300 genes per cell, for replicate one and two, respectively. All data analysis, unless otherwise stated, was performed in R. We excluded outliers with extremely high total UMI counts (1.5 interquartile ranges above the third quartile), cells with less than 3000 total UMI counts, and cells with less than 800 mapped genes. A total of 2671 and 2072 cells, from replicate one and two, respectively, were used for further analysis. Sequencing data are available at GEO: GSE220242

Molecular counts of cells were normalised using size factors calculated with 'scran' package (Lun et al., 2016). Dimensionality reduction was performed using genes with mean normalised UMI counts above 0.01 (9698 genes). Reducing the dimensionality of transcriptome data to two dimensions used principal component analysis (PCA) first, then elastic embedding on the first 11 principal components (selected based on their variance contribution), as described in (Chen et al., 2019). Elastic embedding was performed in MATLAB. For clarity, cells with higher expression are plotted on top of cells with lower expression. The set of aggregation-specific genes is the same as in (Nichols et al., 2020). The genes being up-regulated during the mound stage ("post aggregation") and cell-cycle genes are as in (Antolovic et al., 2019). For all gene sets, we used an additional selection of genes having more than 10 captured transcripts in at least one cell in our dataset, except for cell-cycle genes having more than 1 transcript in at least one cell. The 3D cell density landscape was calculated with the use of 'ks' package in R, using the Hpi (bandwidth estimator) and kde functions. 2D cell density landscapes were plotted with the 'ggplot2' package, with the bandwidth set to 1. The cell-cell correlation matrix and two-way hierarchical clustering were carried in MATLAB, using the clustergram function.

To test the effects of loss of the adenylyl cyclase required for chemotaxis and aggregation (ACA) on the jump, we first identified the sets of genes being repressed at the jump (pre-jump), transiently expressed at the jump (jump), and upregulated after the jump

(post-jump). We established criteria that generated consistent scRNAseq profiles for each category. The cells were divided into pre-jump, jump and post-jump based on the hierarchical clustering of the cell-cell correlation matrix (Fig. S4A). Jump class genes were defined as having fold change (FC) > 3 from before and after the jump, having the mean expression >1 normalised UMIs in the jump region and <4 before. Pre-jump genes were defined as being downregulated twofold in the jump region and tenfold after the jump (compared to the pre-jump region), with mean expression >10 normalised UMIs before and < 3 normalised UMIs after the jump. Post-jump genes were defined as having FC > 10 from before the jump and FC > 0.1 from the jump region, with mean expression < 5 UMIs before and >5 UMIs after the jump. For comparing the behaviour of these sets of genes in populations with or without ACA, we used the population RNAseq data from (Katoh-Kurasawa et al., 2021). For *acaA*$^{-}$ expression values, we used the mean value of both replicates from this published dataset. For wild-type expression values we used mean value of replicates named r5, r6 and r7 (from the published data). We chose these replicates as they were sequenced on Illumina HiSEq 2500, the same as their ACA mutants. The genes we have considered here are those, in wild-type cells, that show the same temporal behaviour in our data (the wild isolate NC4 cells cultured on bacteria) and the earlier study (the lab strain Ax4, cultured in axenic media). A minor fraction of genes in each class (pre-jump 7/61, jump 8/30, post-jump 1/83) did not overlap between the datasets.

Imaging protocols

Cells were harvested from adherent cultures in HL5 medium (Formedium), centrifuged at 720 g for 2 minutes and resuspended in 5 mL KK2. Cells were recentrifuged and resuspended at 1x10^7 cells/mL in KK2. Transcriptional reporter and signalling reporter cells lines were mixed at a 1:2 ratio, and spotted onto an agar plate with diluted SM (1 SM : 19 H_2O) freshly spread with *Klebsiella*. Agar plates were incubated upright in a humid chamber for 3 days when agar pads were excised and inverted onto a 35mm imaging dish (µ-Dish, Ibidi, 81156). Cells were imaged on an inverted spinning disk confocal microscope (3i) using a 63x oil immersion lens, with a Prime 95B CMOS camera (Photometrics). 14 to 16 z slices were acquired with a 0.4 µm step size, with 2x2 binning. 3D stacks were captured every 45 or 60 seconds at multiple xy positions across the cell population. 9 fields of view were stitched together, using Slidebook (3i), to generate a montage of the entire heterogeneous early developmental niche. GFP and mCherry were excited with a 488 nm and 561 nm lasers, respectively, with laser powers optimised for best resolution alongside maintained cell health. Laser power, gain and exposure time were kept consistent between wildtype and *acaA*- cells of the same transcription reporter. Wildtype cells were imaged for 4 hours to capture the full range of cAMP signalling dynamics. The *acaA*- cells, which lack excitable cAMP signalling were imaged for 1.5 hours. For bPAC activation, transcriptional reporter cells were mixed with bPAC expressing cells at a 1:2 ratio. Activation used a 3D stack with a 488 nm laser every 6 or 10 minutes to generate periodic cAMP pulsing. This approach had

the dual function of light activating bPAC and collecting transcription spot data. 12-15 fields of view were captured to sample the extended starvation zone of the *acaA*- cells.

Spot tracking and image analysis

Spot detection was carried out as described (Corrigan et al., 2016) with the modifications that a threshold was imposed to distinguish between spots representing sites of transcription and background. Thresholds were established iteratively by inspection, then the same threshold was used for wild-type and *acaA*- experiments with that transcriptional reporter. Thresholds were applied to the background intensity of the nucleus, discarding very bright and very dim cells, which show non-linear scaling between live transcription site data and smFISH-based intensity measurements. Other modifications to the code from (Corrigan et al., 2016) were exchanging a "for" loop for a "parfor" for parallel computing and changing a parameter ("firad") to cater for the different microscope used in the current study. To plot transcription dynamics in the colony through time and show the broadest dynamic range of the data, values above 3 inter-quartile ranges from the upper quartile were set to the highest colour. Pixels with no detected spots are set to black. To compare wild-type and *acaA*- cells, plots for wild-type and *acaA*- data were set to the same colour scale. To identify cAMP waves, we masked signal from the transcriptional reporter cells, which are more variable in their background intensity than the Flamindo2 cells. The intensity of the remaining cell-containing pixels (representing primarily the Flamindo2 signal) were averaged at each timepoint.

Analysis of spatial signalling and transcription data

To measure jump gene expression, we analysed data from the population before the clear physical separation of the population into starving and streaming cells. The time of population separation was specified manually. Plots showing time series of nuclei counts and spot intensity were produced by plotting nuclear regression fits and spot intensity regression fits for each timepoint. To explore the relationship between cell position, cAMP signalling and jump gene transcription, images were divided into left and right regions, corresponding to recently starved cells and more starved cells. Using R, a local polynomial regression fit (loess function) was applied to nuclei counts across the x-dimension for each timepoint with a span of 0.75. All further local polynomial regression fits (ie. for Flamindo2, spot intensity) were carried out in the same way. The x positions of the minimum of the fit were identified for each timepoint. The range of values of the identified minima positions was used to define the least occupied area, i.e. the approximate location of cell population separation. The region spanning from the edge of the field occupied with bacteria to the edge of the least occupied area was defined as 'left' and the region spanning from the other edge of the least occupied area to the far right of the captured field as 'right'. Mean values of transcription spot intensity and Flamindo2 signal were compared across the left and right regions of the image at each timepoint.

Development • Supplementary information

Inflections in the transcription spot intensity fit for each timepoint were used to characterise the location of cells where gene expression is strongly induced. To capture the dynamic location of the inflection over time, the x coordinate of the inflection was taken, and a local polynomial regression curve fitted linking inflection x coordinates through time, excluding outliers >1.5 interquartile ranges beyond the upper and lower quartiles.

Difference images of Flamindo2 were produced by taking the difference in intensity between one timepoint and the subsequent one, referred to as diff(Flamindo2). As the lowest intensity of Flamindo2 corresponds to the highest intracellular cAMP concentration, the timepoints where cAMP waves pass through the population were found by identifying local minima of mean diff(Flamindo2) in the right-hand region of the image at each timepoint. To find the border of cAMP relay, a local polynomial regression fit was applied to diff(Flamindo2) in x, and inflections in the fit were found for timepoints previously identified as having a cAMP wave. To capture the location of the cAMP signal relay border in time, the x coordinate of the inflection was taken, and a local polynomial regression curve fitted, linking inflection x coordinates through time excluding outliers >1.5 interquartile ranges beyond the upper and lower quartiles.

To investigate the time delays between signalling and transcription, transcription spot intensity mean and Flamindo2 mean signal in the right region of the image were further processed. Using R, both signals initially were locally smoothed using Savitzky–Golay filtering, with a filter length of 3 and a filter order of 1. We then corrected for the changing background over time, by Savitzky–Golay filtering with a filter length of 15 and a filter order of 1. The background was then subtracted from the locally smoothed signal. Flamindo2 signal is inversely related to [cAMP], so to convert Flamindo2 signal into cAMP signal Flamindo2 intensity was inverted. To find the time difference between cAMP and gene transcription dynamics, cross correlation analysis was completed on the smoothed, background corrected signals. Cross correlation was completed over a period of +/- 10 minutes to find realistic signal relay times without artefacts from noisy signal.

References

Antolovic, V., Lenn, T., Miermont, A., Chubb, J.R., 2019. Transition state dynamics during a stochastic fate choice. Development 146.
Chen, Z., An, S., Bai, X., Gong, F., Ma, L., Wan, L., 2019. DensityPath: an algorithm to visualize and reconstruct cell state-transition path on density landscape for single-cell RNA sequencing data. Bioinformatics 35, 2593-2601.
Corrigan, A.M., Tunnacliffe, E., Cannon, D., Chubb, J.R., 2016. A continuum model of transcriptional bursting. eLife 5.
Katoh-Kurasawa, M., Hrovatin, K., Hirose, S., Webb, A., Ho, H.I., Zupan, B., Shaulsky, G., 2021. Transcriptional milestones in Dictyostelium development. Genome research.
Lun, A.T., Bach, K., Marioni, J.C., 2016. Pooling across cells to normalize single-cell RNA sequencing data with many zero counts. Genome biology 17, 75.
Nichols, J.M., Antolovic, V., Reich, J.D., Brameyer, S., Paschke, P., Chubb, J.R., 2020. Cell and molecular transitions during efficient dedifferentiation. eLife 9.

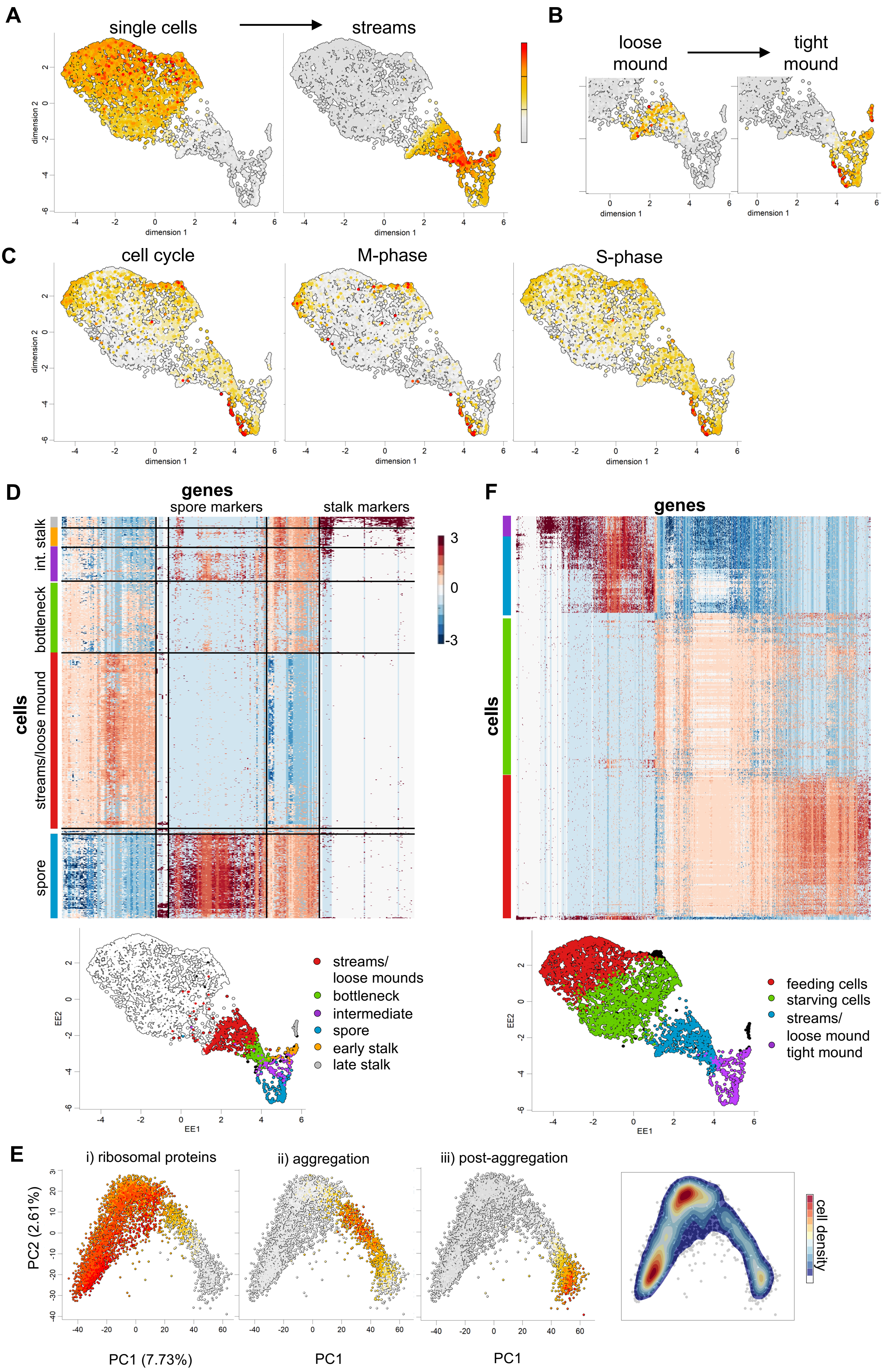

Development • Supplementary information

Fig. S1. Developmental progression in the physiological niche.
A and **B)** Cross referencing the 2D scRNAseq cell map to previously published population RNAseq data: genes identified as markers of major morphological changes in (Katoh-Kurasawa et al., 2021). In A, the genes changing during the transition from single cells to cell streams (down-regulated: 140 genes, up-regulated: 91). In B, the genes changing during the loose to tight mound transition (down-regulated: 4, up-regulated: 83). **C)** Distribution of cell-cycle genes expression on the 2D map (160 genes). For optimal visual contrast, the maximal colour value here is set at mean expression > 0.2. Plots are also shown for M-phase and S-phase genes. The latter shown more diffuse enrichment. **D)** Two-way hierarchical clustering of 1112 cells from the post-jump region (selected as the cells belonging to the right main branch in Fig.S4A). Colour shows z-score values. Genes were selected as having mean normalized expression over 0.01 and being correlated with at least ten other genes with Pearson's |r| > 0.5. Gene clusters expressing spore and stalk markers are indicated at the top of the map. Six main clusters of cells are marked with coloured boxes left of the heatmap. These six clusters are overlaid on the adjacent 2D transcriptome plot. The clusters are streams/loose mounds, bottleneck, intermediate, spore and two stalk populations. **E)** PCA plots labelled with the different stage gene sets used in Figure 1B, showing that the PC1 axis records developmental time. Colours in the far right panel correspond to relative cell density. **F)** Two-way hierarchical clustering of cells described with 957 genes (colour shows z-score values). Genes were selected as in D. Four main clusters of cells are marked with coloured boxes left of the heatmap. These four clusters identified are overlaid on the 2D transcriptome plot. The main clusters are feeding cells (1624), starving cells (1840), streams/loose aggregate (737) and tight aggregate (396). 66% of the identified genes are repressed before the jump.

Development • Supplementary information

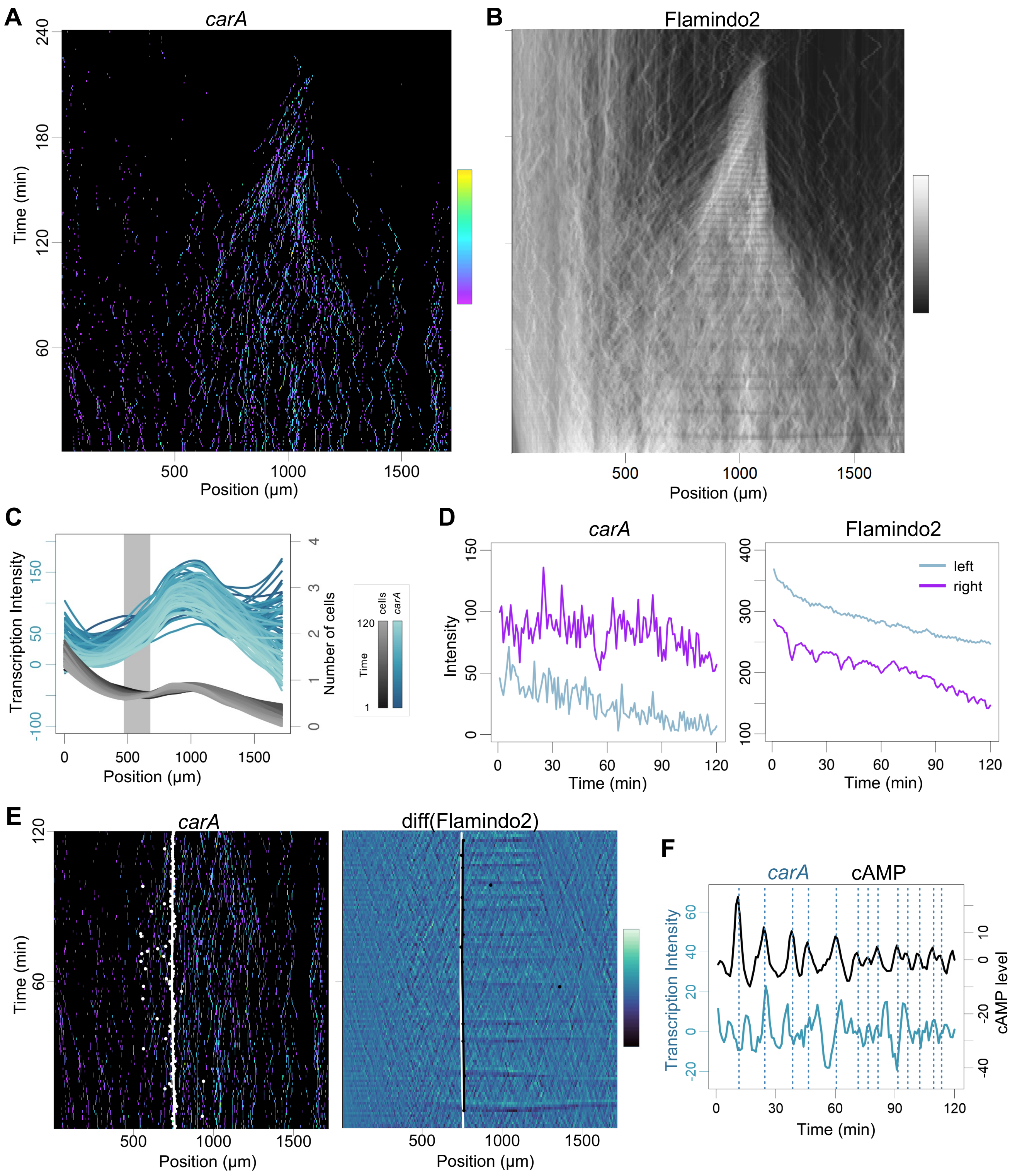
A
carA
240
180
120
60
Time (min)
500
1000
1500
Position (µm)
B
Flamindo2
500
1000
1500
Position (µm)
C
Transcription Intensity
-100
0
50
150
Number of cells
0
1
2
3
4
0
500
1000
1500
Position (µm)
cells
carA
Time
120
1
D
carA
Intensity
0
50
100
150
0
30
60
90
120
Time (min)
Flamindo2
left
right
100
200
300
400
0
30
60
90
120
Time (min)
E
carA
120
60
Time (min)
500
1000
1500
Position (µm)
diff(Flamindo2)
500
1000
1500
Position (µm)
F
carA
cAMP
Transcription Intensity
-20
0
20
40
60
cAMP level
-40
-20
0
10
0
30
60
90
120
Time (min)

Fig. S2. Coupling between jump transcription and signalling dynamics This figure shows a repeat experiment of Figure 3. **A)** Imaging transcription dynamics of the jump gene, *carA*. Horizontal axis reflects the axis of differentiation. Imaging time is represented on the vertical axis. Transcription activity (detected using PP7-PCP) over time is shown, with activity level related by the colour scale on the right. Transcription is initially rare and sporadic, becoming frequent and oscillatory as differentiation proceeds. **B)** Same data as in A, showing cAMP signalling dynamics using the Flamindo2 biosensor, which dims in fluorescence when intracellular [cAMP] increases. Data show oscillations in differentiating cells. Cells merge into an aggregate during the movie. **C)** Increased transcription activity during differentiation. Plots summarises data in B, and also shows the distribution of cells in the population. Changing transcription and cell distributions over time are shown as different colour shades (see colour scale). The grey line corresponds to the minimum in cell density, which corresponds to where the population splits during the transition to multicellularity. **D)** Transitions in transcription and signalling dynamics. Left panel shows the differences in *carA* transcription dynamics between the areas left and right of the grey line in D. Right panel shows the differences between oscillatory and non-oscillatory cAMP dynamics either side of the grey line in D. **E)** Positional coupling between transcription and signalling dynamics. Left- white spots represent inflections of the curves of transcriptional intensity values at each imaging time point. The white line is a regression line summarising the distribution of points. Right- black dots show inflection points for cAMP signalling, where the signal intensity changes at the left extremity of the waves. Black line is the regression summarising these points. White line the same as in left panel. Inflection values were calculated at timepoints corresponding to cAMP wave maxima. **F)** Temporal coupling between transcription and signalling oscillations. Peaks in cAMP signalling (vertical lines) 4-5 minutes prior to peaks in *carA* transcription.

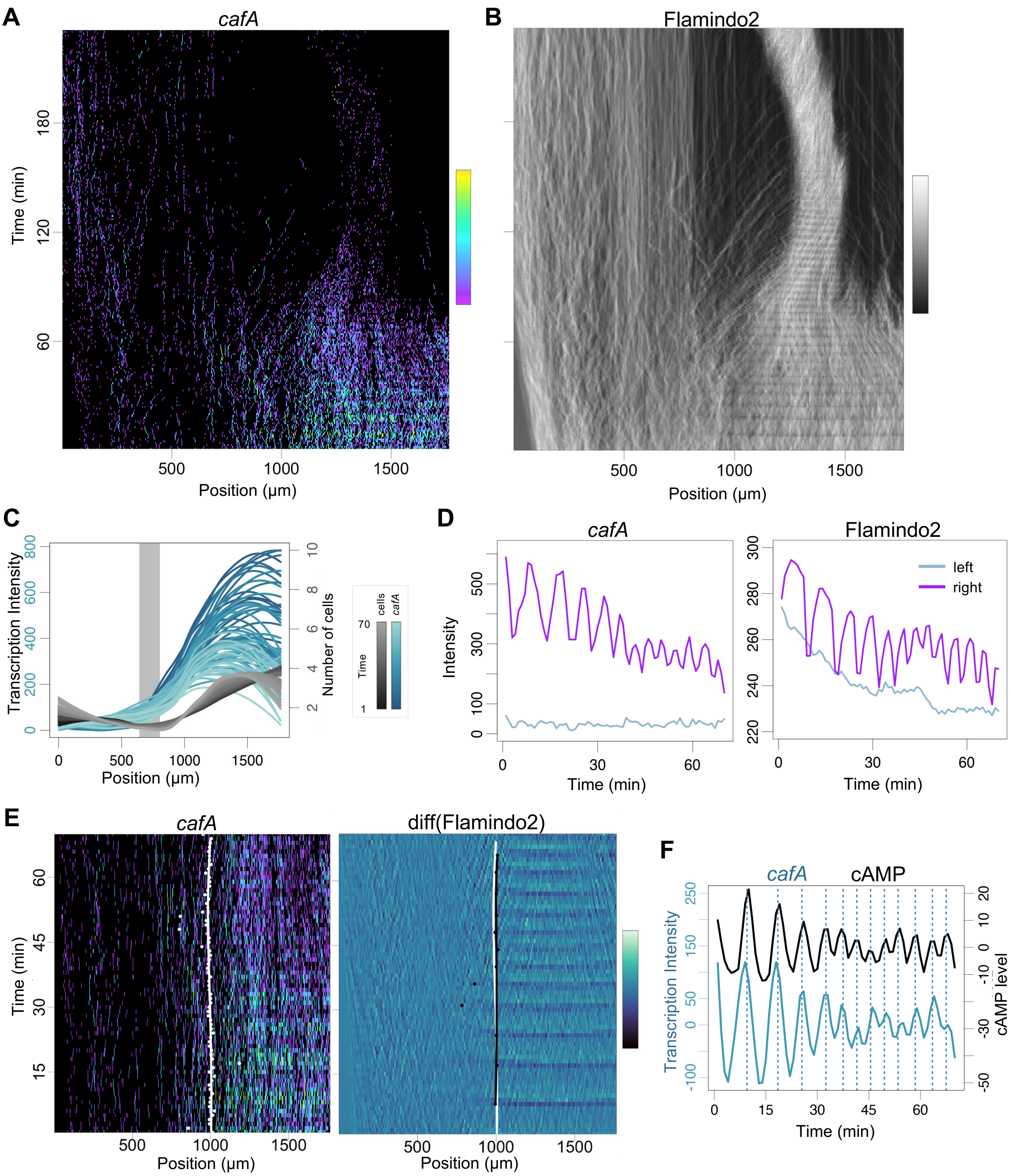

Development • Supplementary information

Fig. S3. Alternative coupling strategies between jump signalling and transcription.
This figure shows a repeat experiment relating to Figure 5.
A) Imaging transcription dynamics of the jump gene, *cafA*. Horizontal axis reflects the axis of differentiation. Imaging time is represented on the vertical axis. Transcription activity measured using the PP7-PCP system. Transcription is initially rare and sporadic, becoming frequent and oscillatory as differentiation proceeds. **B)** Same data as in A, showing cAMP signalling dynamics using Flamindo2. Cells merge into an aggregate during the movie. **C)** Increased transcription activity during differentiation. Plots summarises data in A, and shows the distribution of cells in the population. Changing transcription and cell distributions over time are shown as different colour shades (see colour scale). The grey line corresponds to the minimum in cell density, which corresponds to where the population splits during the transition to multicellularity. **D)** Transitions in transcription and signalling dynamics. Left panel shows the distinction in *cafA* transcription dynamics between the areas left and right of the grey line in C. Right panel shows the distinction between oscillatory and non-oscillatory cAMP dynamics either side of the grey line in C. **E)** Boundaries of transcription and signalling dynamics. Left- white spots represent inflections of the curves of transcriptional intensity values at each imaging time point. The white line is a regression line summarising the distribution of points. Right- black dots show inflection points for cAMP, where the signal intensity changes at the left extremity of the waves. Black line is the regression summarising these points. White line the same as in left panel. Inflection values were calculated at timepoints corresponding to cAMP wave maxima. **F)** Temporal coupling between transcription and signalling oscillations. Peaks in cAMP signalling slightly precede peaks in *cafA* transcriptional activity.

Development • Supplementary information

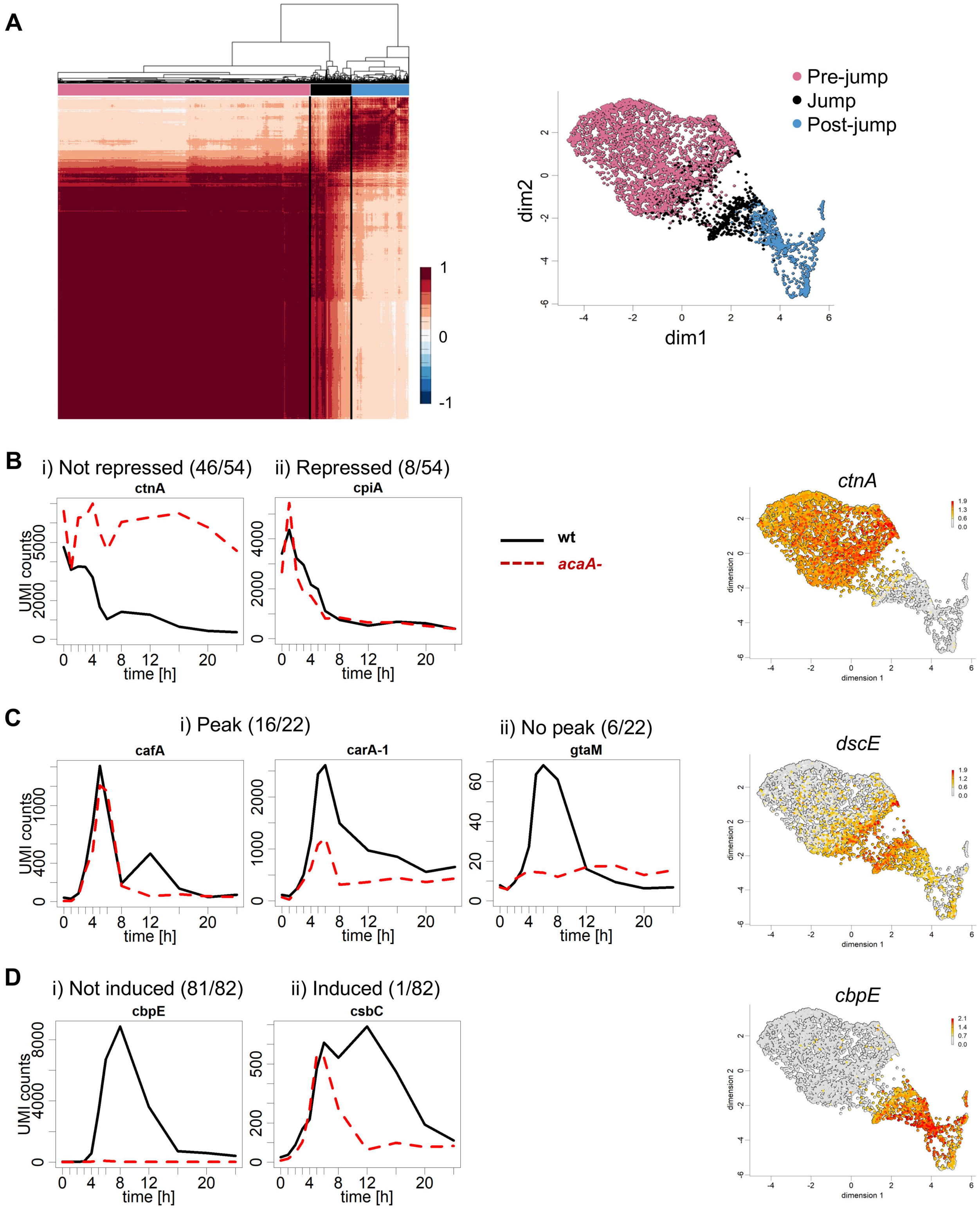

Development • Supplementary information

Fig. S4. Changes in jump gene expression caused by loss of cAMP.
A) Cell-cell correlation matrix of the continuous developmental scRNAseq (the same plot as Fig. 1G, but including the hierarchical tree). We identified three main clusters, with the one in the centre corresponding to the jump. To find jump genes, we expanded this region by taking the most-right subcluster of the left cluster and the most-left subcluster of the right cluster (black box). The modified clusters (before, during and after the jump) marked as (pink, black and blue) are shown in the right panel on the 2D transcriptome plot. We used this division to identify genes downregulated before the jump (**B**), enriched in the jump region (**C**) or upregulated after the jump (**D**). **B – D** show transcript dynamics of genes identified as pre-, during and post-jump, in wild-type cells (black line) and *acaA*$^-$ cells (red dashed line), which lack the aggregation stage cAMP synthesis enzyme. Transcript data from (Katoh-Kurasawa et al., 2021). The proportion of the genes showing each specific behaviour are shown. Also shown are typical examples of scRNAseq expression plots for each class. **B)** i) Most transcripts normally downregulated at the jump retain expression in *acaA*- mutants (85%). ii) A minor proportion of jump repressed transcripts are unperturbed. **C)** i) 73% of jump-specific transcripts retain a peak in expression in *acaA*- mutants, although induction is reduced, ii) 27% of jump specific genes are not induced in *acaA*- cells. **D)** Almost all (99%) genes induced after the jump are not induced in *acaA*$^-$ mutants.

Table S1. The table lists three classes of genes which change their expression around the jump: repressed at the jump (pre-jump genes), induced spanning the jump (“jump” genes) and induced after the jump (post-jump). These gene sets form the basis of the analysis in Supplementary Figure 4.

Available for download at
https://journals.biologists.com/dev/article-lookup/doi/10.1242/dev.201946#supplementary-data

Development • Supplementary information

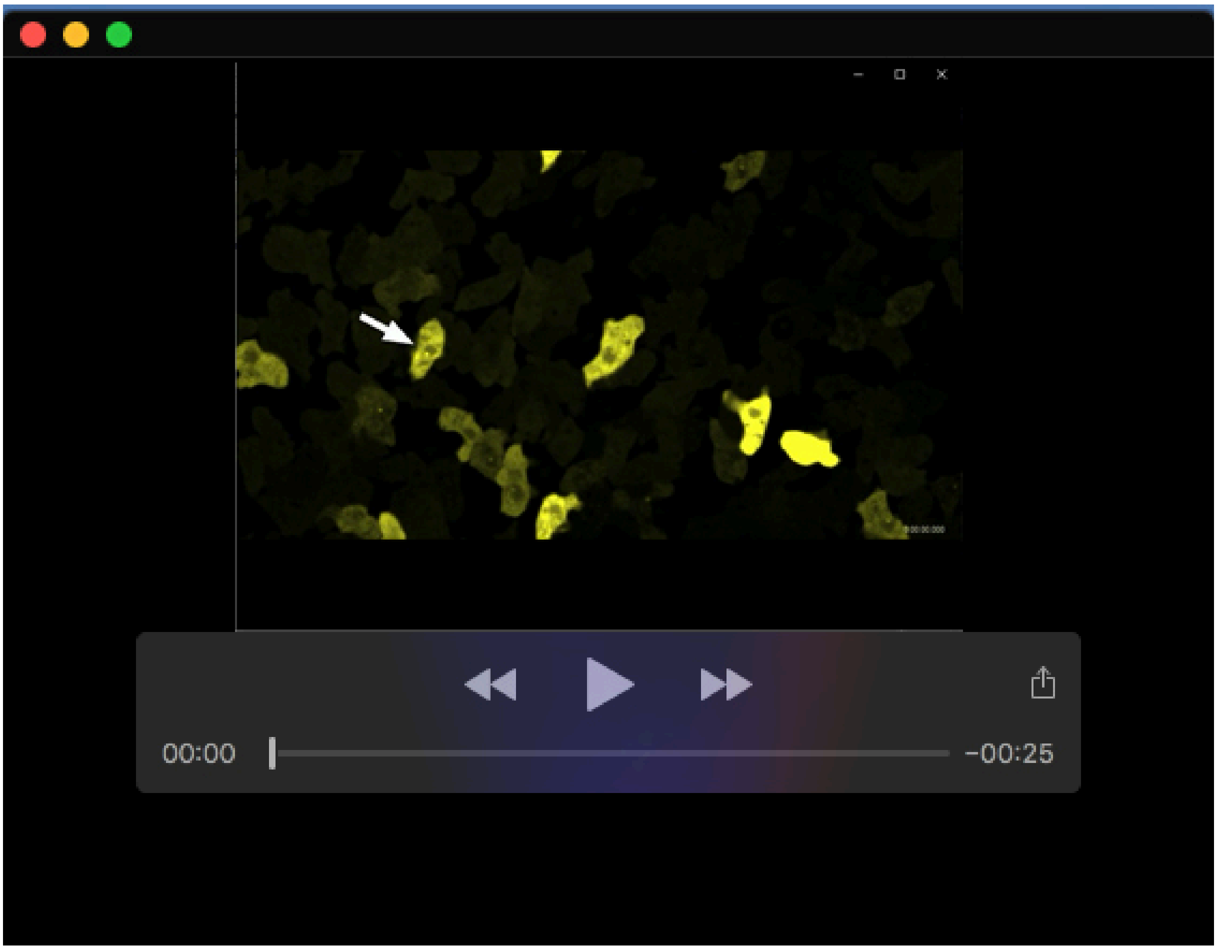

Movie 1. Imaging jump transcription over the length scale of the developmental niche. The time series progressively zooms out, showing transcriptional events in single cells, then zooming out to show the position and movement of individual cells in the context of the early developmental niche. The cells are a mixture of *cafA* transcriptional reporter cells and cells expressing the cAMP reporter, Flamindo2. The contrast has been optimised in this case to make the transcriptional reporter cells clearly visible. In the first frame, the arrow points to a cell with a bright nuclear spot corresponding to nascent *cafA* RNA, at the site of transcription. As the movie zooms out, the full niche becomes apparent, with the undifferentiated cells to the left and the differentiating cells further to the right, with the onset of cell aggregation (streaming) visible. The playback is at 7 frames per second, with each frame captured every 1 minute. When fully zoomed out, the field of view is 1.68mm along the long axis.

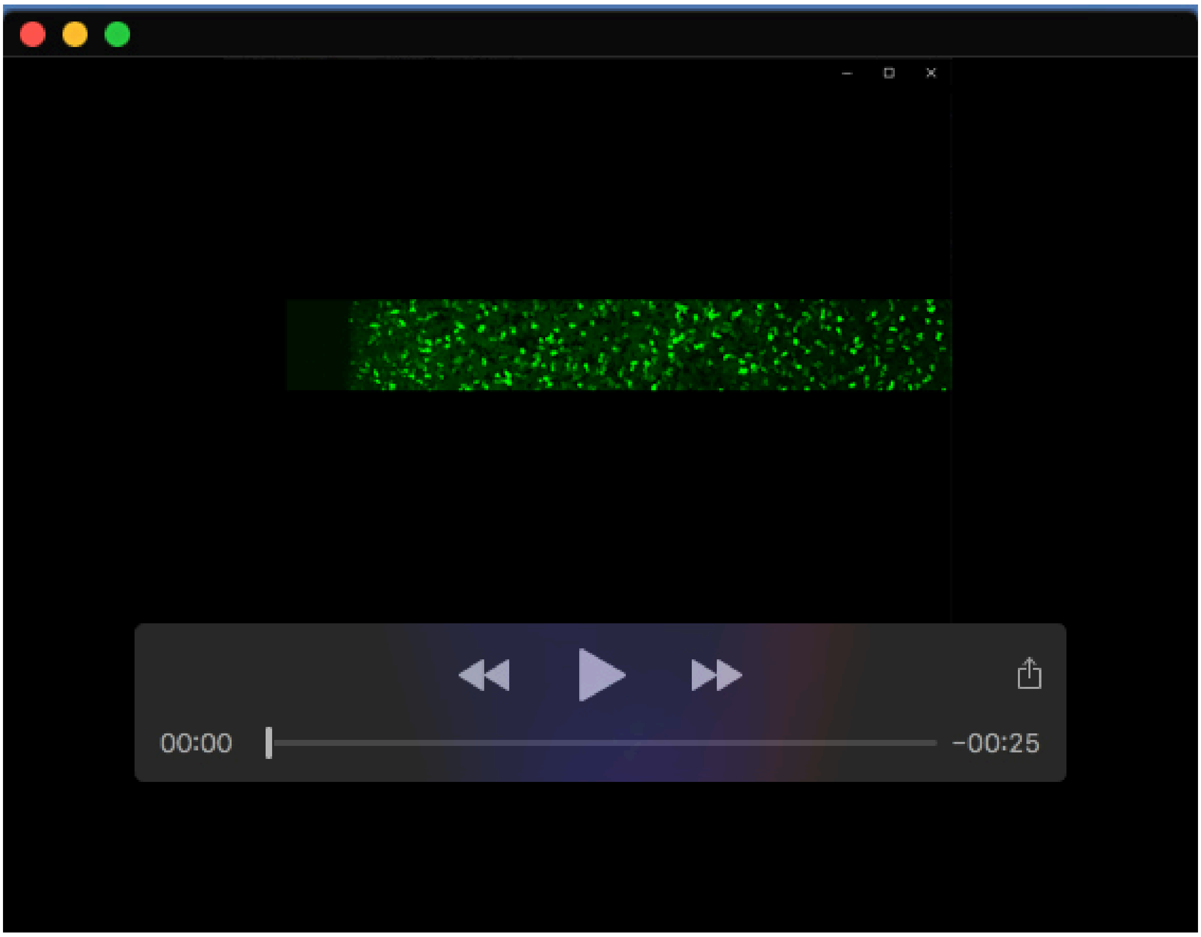

Movie 2. Imaging cAMP signalling dynamics in the developmental niche. This move shows similar data to Movie 1, with the contrast optimised to show the dimmer Flamindo2 expressing cells. The undifferentiated, feeding cells are on the left, with the cell aggregation beginning on the right. Towards the right, the population shows synchronous oscillations in Flamindo2 intensity, corresponding to cAMP oscillations (Flamindo2 signal is inversely related to intracellular cAMP level). The playback is at 15 frames per second, with each frame captured every 45 seconds. The field of view is 1.68mm along the long axis.
