# Supplementary material for: Collective signalling drives rapid jumping between cell states
Source: Development. 2023 Dec 6;150(23):dev201946. doi: 10.1242/dev.201946 (PMC10730084; doi:10.1242/dev.201946)
Supplement: Supplementary file 1 [file develop-150-201946_review_history.pdf]

Collective signalling drives rapid jumping between cell states

Elizabeth R. Westbrook, Tchern Lenn, Jonathan R. Chubb and Vlatka Antolović
DOI: 10.1242/dev.201946

Editor: Paul François

Review timeline

Original submission:	3 May 2023
Editorial decision:	24 July 2023
First revision received:	15 September 2023
Accepted:	19 October 2023

Original submission

First decision letter

MS ID#: DEVELOP/2023/201946

MS TITLE: Collective signalling drives rapid jumping between cell states

AUTHORS: Elizabeth R Westbrook, Tchern Lenn, Jonathan R Chubb, and Vlatka Antolovic

I have now received all the referees reports on the above manuscript, and have reached a decision. The referees' comments are appended below, or you can access them online: please go to BenchPress and click on the 'Manuscripts with Decisions' queue in the Author Area.

The overall evaluation is positive and we would like to publish a revised manuscript in Development, provided that the referees' comments can be satisfactorily addressed. Please attend to all of the reviewers' comments in your revised manuscript and detail them in your point-by-point response. If you do not agree with any of their criticisms or suggestions explain clearly why this is so. If it would be helpful, you are welcome to contact us to discuss your revision in greater detail. Please send us a point-by-point response indicating your plans for addressing the referee’s comments, and we will look over this and provide further guidance.

Reviewer 1

Advance summary and potential significance to field

This paper reports on analysis of gene expression during Dictyostelium development using a combination of single cell RNA sequencing and single cell live transcription imaging for a number of selected genes. The single cell RNA sequencing experiments analyses a mixed population of cells that were growing/developing on a bacterial lawn. These cells represent a cross-section through the Dictyostelium life cycle from vegetive feeding to starving mound/slug stage cells. Expression analysis of >4200 single cells shows that feeding and cells entering starvation are separated from aggregation stage and mound stage cells along the first principal component, which is inferred to indicate the developmental state (time). Since few cells are found in intermediate positions along this axis, this could imply a rather sudden change in gene expression profile during the early starvation to aggregation transition at the single cell level.

Expression of some well-studied key genes like the cAMP receptor, carA and a calcium binding protein, cafA and contact site B, csbA are followed up via single cell transcriptional activity analysis

© 2023. Published by The Company of Biologists under the terms of the Creative Commons Attribution License (https://creativecommons.org/licenses/by/4.0/).

using methods well established in the Chubb lab. Via mapping the onset of expression of these genes on the aggregation field it is shown that cells turn on these genes in the same region that marks the the onset of persistent cAMP signalling, as measured by the Flamindo2 cAMP indicator. These live expression experiments confirm that transcription of these genes is directly coupled to the cAMP oscillations, resulting in clear periodic oscillations of expression. Use of adenylyl cyclase (acaA) null mutant confirms that transcription of carA and cafA is cAMP dependent. carA expression can be rescued in an aca null background though periodic activation of a light-sensitive cyclase, but surprisingly cafA is expression is not Overall these experiments largely confirm earlier understanding of the logic and manner of the differentiation process, but add some significant novel insights/quantitative data on the dynamics of the single-cell differentiation choices.

Comments for the author

There are a number of points that need further clarification
1: The reason behind the jump is not yet quite clear. Some genes necessary for aggregation like the cAMP receptor and possibly caf clearly straddle the jump.
Are there genes that clearly delineate the jump, i.e. genes that are expressed up to the jump and other genes that are expressed immediately after the jump? If so can these be identified and plotted on the cell density map? Alternatively, if no genes can be clearly identified, what then are the characteristic changes in the expression profiles underlie the jump in that case?
2: It is inferred that cells transition from starving to aggregation/mound stage gene expression in a short time span, since only few cells are found to be classified in an intermediate state. On the other hand it is suggested that cells of rather different gene expression status mix into the same structures aggregation stream and mounds (Fig.6), where they are likely exposed to similar signalling conditions. Does this imply that their inherent sensitivity to signals (cAMP?) differs over a wide range, or alternatively that their different history (level of starvation, cell cycle etc) might be important. This maybe difficult to address experimentally in the context of the present study but could be discussed.
3: Differences in cAMP response between caf and car gene expression could be due to differences in requirement for other signals as suggested, but could equally well be due to differences in sensitivity to cAMP signals (amplitude, frequency).
It would be nice to measure cAMP in response to photo-stimulation to find out how much cAMP is actually produced after stimulation and how the dynamics compares to the in-vivo signals.

Minor points
1: The data in fig. 6 are not completely convincing, since they are based on observation of a few cells entering a mound at a late stage (fig 6D). It is not known what differentiation state these cells are in. However more importantly to know whether this is mixing of cells of different developmental times is a major factor, it would be necessary to know whether a significant percentage of cells do so.
2: Data but not tracking and analysis code appears to be available on Github different from what is stated in the text.

<u>Reviewer 2</u>

Advance summary and potential significance to field

The study performs a sequence of biologically informed and physically motivated single-cell expression and imaging measurements. The analysis of the data is elegant and informative. In particular, the study investigates the differentiation of dicty cells along a gradient of starvation. The experiment is performed elegantly, where a move front of dicty cells, feeding on bacteria, "bleb" off differentiating multicellular mounds as nutrients get depleted. This developmental gradient is sampled. Elegant and straightforward analysis of the single-cell gene expression data reveals a transition from growing cells, rich in ribosomal transcripts, towards more differentiated cells -- all the way to fruiting bodies, stalks, and spores. The authors focus on a "jump" in gene expression along this gradient. In particular, at or near the onset of aggregation there appears to be a large discontinuous change in gene expression.

© 2023. Published by The Company of Biologists under the terms of the Creative Commons Attribution License (https://creativecommons.org/licenses/by/4.0/).

This fact is born out not just through the nonlinear embedding of the data, but also PCA and straightforward correlation analyses. The study identifies a small cohort of genes that are expressed on either side, or straddling, the jump region. These genes are visualized using the ms2 live imaging system. The qualitative results of the imaging agree with the inference of the sequencing data -- the "jump genes" indeed are transcriptionally active in the vicinity of the zone where cells are aggregating.

The study hypothesizes that camp is the key signaling molecule that is driving the drastic jump in gene expression in the zone of aggregation. To study this they pair live imaging of a camp reporter system and jump genes. The analysis is elegant and convincing, implicating camp in this transition. A straightforward analysis of a mutant condition is convincing for the neccessaity of camp to drive the phenomena. A straightforward analysis of a optogenetically induced camp also leads us to the same compelling conclusion. The authors end with a very physical result -- an inherently nonlinear feature. The aggregation of cells doesn't happen continuously, as might be expected in a linear way of thinking. Complex nonlinear dynamics lead to the aggregation of cells, and thus there is a disagreement between "absolute time" and "developmental progression".

The authors very elegantly combine this nonlinear reasoning with their overall observations to suggest that a mechanism that combined cell intrinsic starvation cues with more collective cues would be beneficial in the commitment of cells towards differentiated states.

Comments for the author

Feedback:
Small-scale
1. Fig1F PC2 is missing? Can you show it?
2. Fig2B: carA appears twice? Are these just two images?

Opinion
In general I applaud the scientific thrust that permeates this study. So many studies throw resources are doing sequencing and eye catching imaging experiments that amount to few insights. This is not the case here. The authors have both the biology of their system in mind, and questions relating to regulation and differentiation. They use the data to address their questions. I hope the authors follow this study up with a more direct confrontation between a phenomenological model of this system (see https://pubmed.ncbi.nlm.nih.gov/26172740/) to their quantitative data.
I hope the authors leverage this system and their measurements to study the "de-differentiation" of multicellular aggregates once nutrients are resupplied.

First revision

Author response to reviewers' comments

We thank both reviewers for their time and interest in reviewing the manuscript. We are very pleased they found the manuscript interesting and topical. We have responded to their queries with some edits to the text, and we also carried out a further experiment to answer a question raised by Reviewer 1. This has been incorporated into a revised Figure 5F.

Reviewer 1 Comments for the Author:
There are a number of points that need further clarification
1: The reason behind the jump is not yet quite clear. Some genes necessary for aggregation like the cAMP receptor and possibly caf clearly straddle the jump. Are there genes that clearly delineate the jump, i.e. genes that are expressed up to the jump and other genes that are expressed immediately after the jump? If so can these be identified and plotted on the cell density map? Alternatively, if no genes can be clearly identified, what then are the characteristic changes in the expression profiles underlie the jump in that case?

The synthesis of a detailed analysis of how different genes coincide with the jump is shown in

© 2023. Published by The Company of Biologists under the terms of the Creative Commons Attribution License (https://creativecommons.org/licenses/by/4.0/).

Figure S4, with specific examples of the classes of gene expression the reviewer is interested in. There are also panels of genes overlaid on the transcriptome map in Figure 1 and S1 (eg. ribosome genes, aggregation genes etc.). In addition, we have now provided a comprehensive index of the genes that clearly fall into the different classes. This has been placed in a new Supplementary Table 1.

2: It is inferred that cells transition from starving to aggregation/mound stage gene expression in a short time span, since only few cells are found to be classified in an intermediate state. On the other hand it is suggested that cells of rather different gene expression status mix into the same structures aggregation stream and mounds (Fig.6), where they are likely exposed to similar signalling conditions. Does this imply that their inherent sensitivity to signals (cAMP?) differs over a wide range, or alternatively that their different history (level of starvation, cell cycle etc) might be important. This maybe difficult to address experimentally in the context of the present study but could be discussed.

This is a nice point. We have added a sentence in the discussion to expand on this point and to contextualise it with potential effects of heterogeneity on final fate choice.

3: Differences in cAMP response between caf and car gene expression could be due to differences in requirement for other signals as suggested, but could equally well be due to differences in sensitivity to cAMP signals (amplitude, frequency).
It would be nice to measure cAMP in response to photo-stimulation to find out how much cAMP is actually produced after stimulation and how the dynamics compares to the in-vivo signals.

This is an important point. Is the difference between *carA* and *cafA* because of differences in sensitivity to cAMP or something else? Specifically, did *cafA* not respond to optogenetic cAMP because we were not in the correct dynamic range of signalling? To test this idea, we repeated the optogenetic experiment, but using less frequent optogenetic stimulation (every 10 min rather than every 6 min) to mimic early, less frequent and more sporadic cAMP dynamics. These new data show that *cafA* can be induced in this less frequent cAMP stimulation regime, consistent with it being expressed slightly earlier in development. These data have been included as a new panel in Figure 5F and associated text in the manuscript results section. Thanks for the suggestion.

Reviewer 1: Minor points
1: The data in fig. 6 are not completely convincing, since they are based on observation of a few cells entering a mound at a late stage (fig 6D). It is not known what differentiation state these cells are in. However more importantly to know whether this is mixing of cells of different developmental times is a major factor, it would be necessary to know whether a significant percentage of cells do so.

Thanks for pointing this out. We were not clear in the text that Figure 6D is a schematic, not data. This has now been labelled as such in both the text and the legend, and the panel colours changed to make it more distinct from the experimental data in Figure 6B.

2: Data but not tracking and analysis code appears to be available on Github, different from what is stated in the text.

Thanks for pointing this out, the code was based on Corrigan et al 2016, and the modifications to this are now described in the revised text. The bulk of the code posted on Gitbub is new and relates to the spot analysis.

Reviewer 2 Comments for the Author:
Small-scale
1. Fig1F PC2 is missing? Can you show it?

This is shown in Figure S1E, and we have now referred to this in the main text to make the link more clear.

2. Fig2B: carA appears twice? Are these just two images?

© 2023. Published by The Company of Biologists under the terms of the Creative Commons Attribution License (https://creativecommons.org/licenses/by/4.0/).

Thanks, this was not very clear. The cells used for imaging both had two reporters- the lines are 1. cafA-PP7; carA-MS2 and 2. csbA-PP7; carA-MS2. The carA-MS2 is common to both lines, so we show this to provide a benchmark for the relative distributions of gene expression in the developmental niche. We have added a note to the text and Figure legend to explain this, and placed lines to link the pairs of images in the figure.

Opinion
In general I applaud the scientific thrust that permeates this study. So many studies throw resources are doing sequencing and eye catching imaging experiments that amount to few insights. This is not the case here. The authors have both the biology of their system in mind, and questions relating to regulation and differentiation. They use the data to address their questions. I hope the authors follow this study up with a more direct confrontation between a phenomenological model of this system (see https://pubmed.ncbi.nlm.nih.gov/26172740/) to their quantitative data.

This is an interesting thought for a follow up. Thanks.

I hope the authors leverage this system and their measurements to study the "de- differentiation" of multicellular aggregates once nutrients are resupplied.

Also interesting- we have elaborated on this in the discussion. Putting together our data with the literature implies the biochemical changes at jump are not a barrier to de-differentiation, although the morphological consequences of the jump (ie. cell aggregation) do prevent dedifferentiation.

Second decision letter

MS ID#: DEVELOP/2023/201946

MS TITLE: Collective signalling drives rapid jumping between cell states

AUTHORS: Elizabeth R Westbrook, Tchern Lenn, Jonathan R Chubb, and Vlatka Antolovic
ARTICLE TYPE: Research Article

I am happy to tell you that your manuscript has been accepted for publication in Development, pending our standard ethics checks.

Reviewer 1

Advance summary and potential significance to field

See the previous statement

Comments for the author

The authors have provided some interesting additional data and answered my other questions effectively.

© 2023. Published by The Company of Biologists under the terms of the Creative Commons Attribution License (https://creativecommons.org/licenses/by/4.0/).
